# Karyotypic Changes through Dysploidy Persist Longer over Evolutionary Time than Polyploid Changes

**DOI:** 10.1371/journal.pone.0085266

**Published:** 2014-01-09

**Authors:** Marcial Escudero, Santiago Martín-Bravo, Itay Mayrose, Mario Fernández-Mazuecos, Omar Fiz-Palacios, Andrew L. Hipp, Manuel Pimentel, Pedro Jiménez-Mejías, Virginia Valcárcel, Pablo Vargas, Modesto Luceño

**Affiliations:** 1 Molecular Biology and Biochemical Engineering, Pablo de Olavide University, Seville, Spain; 2 Herbarium, The Morton Arboretum, Lisle, Illinois, United States of America; 3 Botany, The Field Museum, Chicago, Illinois, United States of America; 4 Molecular Biology and Ecology of Plants, Tel Aviv University, Tel Aviv, Israel; 5 Biodiversity and Conservation, Real Jardín Botánico CSIC, Madrid, Spain; 6 Systematic Biology, Uppsala University, Uppsala, Sweden; 7 Plant and Animal Biology and Ecology, University of A Coruña, A Coruña, Spain; 8 Biology, Universidad Autónoma de Madrid, Madrid, Spain; CNRS/University Lyon 1, France

## Abstract

Chromosome evolution has been demonstrated to have profound effects on diversification rates and speciation in angiosperms. While polyploidy has predated some major radiations in plants, it has also been related to decreased diversification rates. There has been comparatively little attention to the evolutionary role of gains and losses of single chromosomes, which may or not entail changes in the DNA content (then called aneuploidy or dysploidy, respectively). In this study we investigate the role of chromosome number transitions and of possible associated genome size changes in angiosperm evolution. We model the tempo and mode of chromosome number evolution and its possible correlation with patterns of cladogenesis in 15 angiosperm clades. Inferred polyploid transitions are distributed more frequently towards recent times than single chromosome gains and losses. This is likely because the latter events do not entail changes in DNA content and are probably due to fission or fusion events (dysploidy), as revealed by an analysis of the relationship between genome size and chromosome number. Our results support the general pattern that recently originated polyploids fail to persist, and suggest that dysploidy may have comparatively longer-term persistence than polyploidy. Changes in chromosome number associated with dysploidy were typically observed across the phylogenies based on a chi-square analysis, consistent with these changes being neutral with respect to diversification.

## Introduction

While variation in chromosome number is widespread among plants, its role in species diversification has long been debated [1]–[3]. Transitions in chromosome number comprise the multiplication of a whole chromosome set (which may entail a whole genome duplication, WGD, or an increase by half of the genome, demipolyploidy, which can occur when one homeologous set of chromosomes is duplicated in an already existing polyploid [4]), and changes in single chromosome number (resulting from fission, fusion, duplication or deletion of single or few chromosomes; [5]). Demipolyploidy is thought to occur through the crossing of gametes of different ploidy levels (e.g., a tetraploid crossed with a diploid, followed or preceded by a doubling of the genome, yields a hexaploid, which is inferred as a 4× to 6× demiploid transition). Researchers have long argued about the prevalence of polyploidy in flowering plants as well as its evolutionary and genomic consequences [3]. Recent works have demonstrated that polyploidy is ubiquitous in angiosperms and has played an important role in many lineages, with evidence of several rounds of both ancient and recent polyploidizations [6]–[11]. The terms paleo- and neopolyploid have been loosely used to describe the relative time of the polyploidy event - either as ancient (paleo) or recent (neo). However, what is ancient and recent varies among authors (e.g., neopolyploidy may be defined as a polyploidization event within a genus or within a species; [12]). In addition, there are still conflicting opinions on whether there is a positive or negative relationship between ancient polyploid events and diversification rates. Whereas polyploids have traditionally been regarded as evolutionary dead ends [1], [13]–[14], recent studies have suggested correspondence between polyploid events and diversification of some of the most species-rich angiosperm lineages [3], [15]. Polyploidizations are rather frequent, but newly formed polyploid lineages generally fail to persist, which would explain their low diversification rates and the biased distribution of polyploidy towards terminal branches of the plant tree of life [10], [17]. Whereas neopolyploids are widespread among angiosperms (an estimated ca. 30% of extant species have polyploidized since their genus arose [8], [10]), paleopolyploidization events are comparatively very rare [9], [11], [17]. Thus, although polyploidy may initiate rapid diversification within distinct evolutionary lineages (i.e. seed plants and angiosperms [9]), it is generally associated with decreased diversification rates and higher extinction risk [8], [10]. Polyploid lineages may have succeeded in becoming established during past periods of great environmental upheaval and mass extinction events, which created new ecological niches and disturbed habitats [17]–[19]. The surviving polyploid lineages could have an enhanced potential for diversification due to their genetic, genomic and epigenetic features [2], [11], [16], [20]–[24], thus attaining long-term evolutionary success [10]–[11], [17], [19]. A parallel situation may currently be depicted by the high proportion of polyploid species in harsh environments like those in high altitudes or latitudes [25]–[26].

In this study, we consider gains and losses of single chromosomes as processes that entail (i) change in DNA content (aneuploidy: duplication or losses of chromosomes), or (ii) little or negligible change in DNA content (dysploidy: fission and/or fusion; Supporting Information S1). Despite the prevalence of gains and losses of single chromosomes among angiosperms [27], the evolutionary role of these chromosome number transitions has received far less attention than that of polyploidy. Some studies have suggested an important role of gains and losses of single chromosomes in species diversification [28]–[33], but there has been no formal test of this role.

A central issue in the study of chromosome number evolution concerns the relative importance of polyploid transitions versus gains and losses of single chromosomes [24]. While polyploidy takes shape as a more dramatic transition – one that involves the whole genome – in practice, the persistence of polyploid lineages is widely believed to be higher compared to aneuploid ones [24]. In contrast, to our knowledge, the evolutionary persistence of lineages affected by dysploidy has not been studied.

Here, we perform a phylogenetic comparative analysis of chromosome evolution and lineage diversification in 15 flowering plant clades to estimate the relative importance of polyploidy and gains and losses of single chromosomes in the evolution of angiosperms. The particular aims of this study are to evaluate (i) previous hypotheses about the role of polyploidy in angiosperm diversification; (ii) the persistence of gains and losses of single chromosomes (including a priori aneuploidy and dysploidy) along angiosperm evolution; and (iii) the relative distribution and timing of polyploidy and gains and losses of single chromosomes across angiosperm phylogenies.

## Materials and Methods

### Phylogenetic sampling

We used molecular phylogenies from 15 angiosperm groups (Supporting Information S1; Material S1) belonging to both dicots (10) and monocots (5), and displaying both monocentric and holocentric chromosomes (Table 1; Supporting Information S1). The four Cyperaceae datasets included in this study were treated independently: the Cariceae dataset represents the main lineages of the tribe and comprised 1–2 species per each *Carex* section, whereas the three *Carex* sections (*Ovales*, *Phacocystis*, and *Spirostachyae*) included a high percentage of the extant species of each of those sections. Each co-author provided 1–2 datasets for which they were experts, and which had a reliable published phylogeny, information to calculate absolute times of divergence and chromosome number variation (Supporting Information S1; Material S1). Taxonomic level of the phylogenies was heterogeneous, comprising families (two), tribes (three), genera (five) and sections (five). Molecular phylogenies with branch lengths scaled to the number of nucleotide substitutions were obtained from the authors of the original published studies (Supporting Information S1; Material S1). The trees were rooted using outgroup species or clades as specified in the original studies and dated using the penalized likelihood method [34] as implemented in the APE R package [35]–[36]. The most appropriate smoothing parameter was chosen based on an initial cross-validation run and calibration points were included to transform trees from relative to absolute times. For the Orchidinae dataset, an ultrametric tree previously generated using BEAST [37] was directly obtained from the study author (Supporting Information S1; Material S1). Chromosome counts (used for the reconstruction of chromosome number transitions) were obtained for each group from different sources (Supporting Information S1; Material S1). Tips of the phylogeny without available chromosome counts were pruned. All our datasets had: (i) sequence data for at least 12 species (maximum 100 species); (ii) chromosome counts available for at least 75% of the species sampled in the phylogeny, and (iii) variation in chromosome numbers among the species remaining in the phylogeny (Table 1).

**Table 1:** Main features of the datasets analysed in this study.

Focal group (order, family)	Species richness	S_phy_ *	S_counts_ *	2*n*-range*	Centromeretype
*Hedera* (Apiales, Araliaceae)	12	12	12	24 – 96	Monocentric
Orchidinae (Asparagales, Orchidaceae)	ca. 1800	103	73	10–82	Monocentric
*Bellis*, *Bellium*, *Bellidastrum* (Asterales, Asteraceae)	21	19	18	9 – 45	Monocentric
*Helianthus* (Asterales, Asteraceae)	49	47	47	17 – 51	Monocentric
Resedaceae (Brassicales)	ca. 85	66	35	6 – 40	Monocentric
*Arenaria* sect. *Plinthine* (Caryophyllales, Caryophyllaceae)	14	14	14	9 – 70	Monocentric
*Erodium* (Geraniales, Geraniaceae)	ca. 74	66	55	8 – 80	Monocentric
Antirrhineae (Lamiales, Plantaginaceae)	ca. 326	44	36	6 – 18	Monocentric
*Passiflora* (Malphigiales, Passifloraceae)	ca. 530	61	56	6 – 12	Monocentric
Cistaceae (Malvales)	ca. 180	47	45	5 – 24	Monocentric
Cariceae (Poales, Cyperaceae)	ca. 2000	135	100	6 – 57	Holocentric
*Carex* sect. *Ovales* (Poales, Cyperaceae)	ca. 90	57	57	26 – 43	Holocentric
*Carex* sect. *Phacocystis* (Poales, Cyperaceae)	ca. 70	35	21	30 – 46	Holocentric
*Carex* sect. *Spirostachyae* (Poales, Cyperaceae)	ca. 70	38	25	30 – 42	Holocentric
*Saxifraga* sect. *Saxifraga* (Saxifragales, Saxifragaceae	ca. 70	56	50	8 – 110	Monocentric

S_phy_: number of species sampled in the phylogeny; S_counts_: number of species sampled in the phylogeny with known chromosome counts; C-range: observed range of chromosome numbers.

### Tempo and mode of chromosome evolution

Given the dated molecular phylogenies and the assignments of chromosome numbers to the tips, we aimed to infer the location of chromosome number transitions using the ChromEvol methodology [4]. This likelihood-based method assesses the fit of several models that allow for various types of chromosome number change along the phylogenies and infers the type of each transition in chromosome number (WGD, demipolyploidy, increase and decrease in a single chromosome; Table 2) along the branches of the tree. We ran all eight available models and used the Akaike information criterion (AIC [38]) to select the best model for each dataset. Models of chromosome evolution in ChromEvol are based on two (gain and loss of a single chromosome) to six parameters (polyploidy, demipolyploidy, gain and loss of a single chromosome and gain and loss of a single chromosome proportional to the current chromosome number) representing chromosome transitions (Table 2). The expected numbers of polyploidy events and gains and losses of single chromosomes along each branch of the phylogeny were recorded based on the best-fitting model.

**Table 2:** Best-fitting model of chromosome number evolution, basic chromosome number (x) at the root of the tree with its probability (in brackets), and inferred number of chromosome gains, losses, polyploidy (PP), and demipolyploidy (Demi-PP) events for each dataset.

Focal group (order, family)	Best supported model*	x (P>0.05)	Gains	Losses	PP	Demi-PP	P value from Chi-square for Polyploidy**	P value from Chi-square for gains and losses of single chromosomes**	Type of gains and losses of single chromosomes
*Hedera* (Apiales, Araliaceae)	CRD	24 (0.92)	0	0	2	5	0.2255 (obs recent > exp recent, obs ancient < exp ancient)	-	-
Orchidinae (Asparagales, Orchidaceae)	CRD	21 (0.88), 22 (0.11)	1	32	3	1	0.0084 (obs recent > exp recent, obs ancient < exp ancient)	0.3477 (obs recent > exp recent, obs ancient < exp ancient)	Dysploidy
*Bellis*, *Bellium*, *Bellidastrum* (Asterales, Asteraceae)	CRDE	9 (0.99)	0	0	6	0	0.0028 (obs recent > exp recent, obs ancient < exp ancient)	-	-
*Helianthus* (Asterales, Asteraceae)	CRD	17 (0.96)	0	0	11	11	**0.0001 (obs recent > exp recent, obs ancient < exp ancient)**	-	-
Resedaceae (Brassicales)	CRDE	3 (0.256), 4 (0.25), 2(0.20), 5 (0.15), 1 (0.08), 6 (0.05)	21	0	10	1	0.3140 (obs recent > exp recent, obs ancient < exp ancient)	0.7685 (obs recent < exp recent, obs ancient < exp ancient)	Dysploidy
*Arenaria* sect. *Plinthine* (Caryophyllales, Caryophyllaceae)	LR	None P>0.05	920	1270	46	0	**0.0004 (obs recent > exp recent, obs ancient < exp ancient)**	0.7384 (obs recent > exp recent, obs ancient < exp ancient)	Undetermined
*Erodium* (Geraniales, Geraniaceae)	CRDE	10 (0.45), 9 (0.36), 5 (0.10)	2	3	9	1	**0.0001 (obs recent > exp recent, obs ancient < exp ancient)**	**0.0001 (obs recent > exp recent, obs ancient < exp ancient)**	Undetermined
Antirrhineae (Lamiales, Plantaginaceae)	LRDE	9 (0.96)	0	14	6	1	0.5354 (obs recent < exp recent, obs ancient > exp ancient)	0.5866 (obs recent < exp recent, obs ancient > exp ancient)	Dysploidy
*Passiflora* (Malphigiales, Passifloraceae)	CRD	6 (0.99)	1	0	4	2	0.3355 (obs recent < exp recent, obs ancient > exp ancient)	0.7407 (obs recent > exp recent, obs ancient < exp ancient)	Dysploidy
Cistaceae (Malvales)	CR	4 (0.87), 2 (0.08)	7	0	9	0	0.1743 (obs recent > exp recent, obs ancient < exp ancient)	0.3006 (obs recent > exp recent, obs ancient < exp ancient)	Dysploidy
Cariceae (Poales, Cyperaceae)	LR	None P>0.05	3480	3699	3	0	**0.0001 (obs recent > exp recent, obs ancient < exp ancient)**	0.0269 (obs recent > exp recent, obs ancient < exp ancient)	Dysploidy
*Carex* sect. *Ovales* (Poales, Cyperaceae)	LRND	None P>0.05	1176	1086	0	0	-	0.9765 (obs recent > exp recent, obs ancient < exp ancient)	Dysploidy
*Carex* sect. *Phacocystis* (Poales, Cyperaceae)	CRND	38 (0.32), 37 (0.23), 39 (0.23), 36 (0.09), 40 (0.08)	74	101	0	0	-	0.2936 (obs recent >exp recent, obs ancient < exp ancient)	Dysploidy
*Carex* sect. *Spirostachyae* (Poales, Cyperaceae)	CRND	38 (0.27), 39(0.24), 37 (0.19), 40 (0.13), 36 (0.08)	12	34	0	0	-	0.3439 (obs recent > exp recent, obs ancient < exp ancient)	Dysploidy
*Saxifraga* sect. *Saxifraga* (Saxifragales, Saxifragaceae	CRD	25 (0.08), 24 (0.08), 26 (0.08), 23 (0.08), 27 (0.07), 22 (0.07), 28 (0.06), 21 (0.06), 29(0.05), 20 (0.05)	0	216	29	0	0.4278 (obs recent > exp recent, obs ancient < exp ancient)	0.1471 (obs recent < exp recent, obs ancient > exp ancient)	Dysploidy

Significant P values from the Chi-square analyses for the present to 10% of total time (“recent” times) vs. rest of the chronogram tree (“ancient” times) temporal level are shown, comparing the number of observed (inferred by ChromEvol analysis) vs. expected (under the null hypothesis of constant transition rate through time) polyploidy and gains/losses transitions. The type of gains and losses of single chromosomes has been inferred from the bibliography (Supporting Information S1 Notes S2) and from the results of the analysis of the relationship between chromosome number and genome size (Supporting Information S2).

CR =  Constant_Rate, three parameters: gains and losses of single chromosomes and polyploidy; CRD =  Constant_Rate_Demi, three parameters: gains and losses of single chromosomes and one for polyploidy and demipolyploidy; CRDE =  Constant_Rate_Demi_Est, four parameters: gains and losses of single chromosomes, polyploidy and demipolyploidy; CRND =  Contant_Rate_No_Dupli, two parameters: gains and losses of single chromosomes; LR =  Linear_Rate, five parameters: gains and losses of single chromosomes, gains and losses of single chromosomes proportional to chromosome number, and polyploidy; LRD =  Linear_Rate_Demi, five parameters: gains and losses of single chromosomes, gains and losses of single chromosomes proportional to chromosome number, and one for polyploidy and demipolyploidy; LRDE =  Linear_Rate_Demi_Est, six parameters: gains and losses of single chromosomes, gains and losses of single chromosomes proportional to chromosome number, polyploidy and demipolyploidy; and LRND =  Linear_Rate_No_Dupli, four parameters: gains and losses of single chromosomes and gains and losses of single chromosomes proportional to chromosome number.

Alternatives analyses (internal vs. external branches of the chronogram tree, present to 25% of total time vs. rest of the chronogram tree, and present to 50% of total time vs. rest of the chronogram tree) reveal identical conclusions.

Using the ChromEvol software (evaluatePPDist option), we calculated the observed chromosome number transitions per unit of time that occurred relatively recently (four temporal strategies: tip branches, from present to 10% of total time, from present to 25% of total time and from present to 50% of total time) and those that occurred deeper in time (the rest of the tree). We calculated the expected number of each type of chromosome number transition (polyploidizations plus demipolyploidizations and gains plus losses of single chromosomes) along the tree assuming that they occur homogenously over time as the null hypothesis (total number of events inferred along the tree divided by the total time). Then chi-square was used to test whether the number of observed transitions along external and internal branches for each temporal level is significantly different than the number of transitions under the expectation of constant transition rate along the tree (null hypothesis). P values smaller than 0.002 were considered significant to reject the null hypothesis. We selected this conservative P-value because our tests are two-tailed and because we did multiple tests and therefore Bonferroni correction is required. Nevertheless, P-values smaller than 0.025 were considered as marginal support to reject the null hypothesis.

### Study of gains and losses of single chromosomes

Gains and losses of single chromosomes encompass different phenomena with various expected outcomes: 1) Aneuploidy: duplication or loss of a chromosome including its DNA content, and 2) Dysploidy: chromosome fusion/fission that do not result in changes in DNA content. In addition, losing chromosomes after polyploidization has a different outcome than when there is not previous polyploidization (in the first case, although genes may be lost, there are extra copies of the genes). In order to differentiate between different patterns of gains and losses of single chromosomes for lineages for which these transitions were inferred, we gathered genome size values from the Plant DNA C-values database (data.kew.org/cvalues/) and references therein. We then analyzed the possible correlation between genome size and chromosome numbers (excluding species affected by polyploidy) both visually and using linear models as implemented in the function *lm* of the software R [36]. These analyses were not performed for lineages with holocentric chromosomes (Cariceae and other *Carex* datasets) since gains and losses of single chromosomes in these groups is already known to be from fissions and fusions (dysploidy; see Supporting Information S1 [39]–[41]). We also analyzed the chromosome number reconstructions obtained from ChromEvol to classify losses of single chromosomes as occurring following a polyploidization event or not.

## Results

The studied groups comprise a high cytogenetic variability, with chromosome numbers ranging from *n* = 5 to *n* = 110 (Table 1). ChromEvol analyses (Table 2, Table S1 in Supporting Information S2) reveal that models of chromosome evolution including polyploidy (12 groups) are more frequent than those which do not (three groups). Interestingly, demipolyploidization, associated with hybridization between different ploidy levels and allopolyploidization processes [4], is inferred in seven of them. Gains and losses of single chromosomes are inferred in 12 groups: 10 of them are affected by single chromosome gains, nine by losses and seven by both events. Rates of gains and losses of single chromosomes are independent of the current chromosome number except for four groups (see inferred models in Table 2). We have found a high heterogeneity in chromosome transition rates both for gains and losses of single chromosomes (0.0013–80.9440 #transitions per million year (my^−1^)) and polyploid transitions (0.0013–0.7888 #transitions my^−1^) (Supporting Information S1). The absolute number of chromosome gain and loss events inferred by our analyses is much higher than that of polyploidy for all groups with both types of transitions, with the exception of *Passiflora*, *Erodium* and Cistaceae datasets (Table 2).

Our analyses (at four different temporal levels) comparing the expected and the observed number of transitions per time reveal similar results: First, the number of polyploid transitions towards the tips of the trees is significantly higher than expected under the null hypothesis (constant polyploidization rate through time) in four of the 12 datasets with polyploidy, in two data sets there is marginal support to reject the null hypothesis whereas for the remaining six data sets the results were congruent with it (Table 2). Second, gains and losses of single chromosomes were in general distributed much more evenly across the trees, with ten of the 12 datasets exhibiting a distribution of gains and losses of single chromosomes statistically indistinguishable from expectation under the null hypothesis (constant chromosome gains and losses transition rate), whereas one exhibited a significantly higher number of these events towards the tips (*Erodium*, Table 2) and one (Cariceae) displayed marginal support for the latter pattern (but with opposite results when comparing separately gains and losses; results not shown). Correlation analyses between genome size and chromosome numbers (6 of 12 datasets; Tables S2–S7 and Figures S1–S6 in Supporting Information S2) and previous studies for the Cyperaceae revealed that gains and losses of single chromosomes are probably due to dysploidy for at least 10 of the 12 datasets for which this kind of transition was inferred (Table 2, Tables S2–S7 and Figures S1–S6 in Supporting Information S2). Therefore, dysploid transitions occurring in these groups would mostly correspond to fissions, fusions and genome rearrangements without changes in DNA content (Table 2; Supporting Information S1). For the remaining two datasets (*Arenaria* and *Erodium*) we cannot differentiate between dysploidy and aneuploidy.

## Discussion

In this study, we have compared the expected vs. observed number of chromosome transitions in a temporal context following several alternative strategies (tips vs. rest of the chronogram tree, present to 10% of total time vs. rest of the chronogram tree, present to 25% of total time vs. rest of the chronogram tree, and present to 50% of total time vs. rest of the chronogram tree) obtaining identical results. Inferred polyploidizations were distributed closer to the tips of the trees than expected under constant chromosome transition rate (null hypothesis) for half of datasets under all tests (Table 2). The cited pattern was significant for four of 12 datasets, and for two additional datasets the analyses suggest marginal support against null hypothesis. In other words, ancient polyploidy events are underrepresented within the half angiosperm clades studied here. Presuming that the rate of polyploidy is constant within lineages, this result suggests that polyploidy rarely has long-term evolutionary success [9]–[11], [17]. Alternatively, the methodology implemented in ChromEvol could have lower power to detect ancient polyploidy events than recent ones. However, studies based on other approaches [9], [11], [17] have reached similar conclusions. Our results support the general pattern that recently originated polyploids fail to persist and diversify at lower rates [10]. Our data provide no obvious insight into the fact that some of the most extensive plant radiations have been predated by a polyploidization [3], [9], [17].

Gains and losses of single chromosomes may originate via many disparate mechanisms, which entail small changes in the chromosome number and changes (aneuploidy) or not (dysploidy) in DNA content (Supporting Information S1). Gains and losses of single chromosomes are widespread among angiosperms [26], and imply far less genomic disruption than polyploidy (it only affects one or a few chromosomes rather than a complete chromosome set and it does not necessarily entail changes in DNA content; Supporting Information S1). However, polyploidy is much more studied and often viewed as the most common type of chromosome transition in plants and the main chromosomal driver of plant diversification [12]. Our results indicate that gains and losses of single chromosomes are also very common across angiosperms (12 of 15 groups) and that they may frequently co-occur with polyploidy within lineages (both kind of transitions inferred in nine of 15; Table 2).

Our results suggest that most of the inferred gains and losses of single chromosomes are dysploid events, and therefore these transitions mostly correspond to changes in chromosome number without changes in DNA content. Gene balance theory predicts that loss or duplication of a subset of chromosomes (aneuploidy) should be more strongly selected against than whole-genome duplication [24]. In congruence, as far as we are concerned, in contrast to polyploidy, ancestral aneuploidy has not been inferred in angiosperms. Nonetheless, our results demonstrate that dysploidy is very frequent in angiosperms, and its effects on lineage diversification deserve further study. Observed dysploid events were not distributed closer to the tips of the phylogeny than expected for most datasets (Table 2). Therefore, contrary to polyploidy, dysploidy appears to be equally distributed early and late across the phylogenies examined. This is consistent with the hypothesis that fusion and fission events are neutral with respect to long-term diversification processes, neither increasing nor decreasing speciation and extinction processes substantially.

Interestingly, holocentric chromosomes (without localized centromere) evolve almost strictly by fission and fusion which do not convey changes in DNA content and those rearrangements are generally neutral or nearly so because of the effects of the diffuse centromere [41]. Moreover, structural changes from fission and fusion are expected not to be underdominant [40]. Our results are in line with this hypothesis for the four datasets with holocentric chromosomes as we cannot reject the null hypothesis for any of them (Table 2). Additional studies are required to compare diversification patterns of organisms having different kinds of chromosomes (holocentric vs. monocentric chromosomes; Supporting Information S1).

To sum up, our results support the hypothesis that dysploidy is less disadvantageous than polyploidy in terms of generating long term persisting lineages [17]. This is most likely because the inferred dysploid transitions typically do not necessarily entail changes in DNA content but only genome structural rearrangements (Supporting Information S1).

## Supporting Information

Supporting Information S1 A. Glossary of cytogenetic terms used throughout the article. B. Results and discussion concerning the individual datasets used for our study.
(DOCX)

Supporting Information S2 Table S1. Parameters of the best supported model inferred for each group with ChromEvol. Table S2. Species, diploid chromosome number (*2n*) and genome size (2C, pg) for Cistaceae. Table S3. Species, diploid chromosome number (*2n*) and genome size (2C, pg) for Antirrhineae. Table S4. Species, diploid chromosome number (*2n*) and genome size (2C, pg) for *Saxifraga* sect. *Saxigraga*. Table S5. Species, diploid chromosome number (*2n*) and genome size (2C, pg) for *Orchidinae*. Table S6. Species, diploid chromosome number (*2n*) and genome size (2C, pg) for Resedaceae. Table S7. Species, diploid chromosome number (*2n*) and genome size (2C, pg) for *Passiflora*. Figure S1. Linear model for the correlation between diploid chromosome number (*2n*) and genome size (2C, pg) in Cistaceae. Figure S2. Linear model for the correlation between diploid chromosome number (*2n*) and genome size (2C, pg) in Antirrhineae. Figure S3. Linear model for the correlation between diploid chromosome number (*2n*) and genome size (2C, pg) in *Saxifraga* sect. *Saxigraga*. Figure S4. Linear model for the correlation between diploid chromosome number (*2n*) and genome size (2C, pg) in *Orchidinae*. Figure S5. Linear model for the correlation between diploid chromosome number (*2n*) and genome size (2C, pg) in Resedaceae. Figure S6. Linear model for the correlation between diploid chromosome number (*2n*) and genome size (2C, pg) in *Passiflora*.
(DOCX)

Material S1 Zip file with graphs of phylogenetic trees with haploid chromosome numbers in the tips and phylogentic trees in parenthetical format from chromEvol analyses with inferred mutation events.
(ZIP)
