# Supplementary material for: Karyotypic Changes through Dysploidy Persist Longer over Evolutionary Time than Polyploid Changes
Source: PLoS One. 2014 Jan 9;9(1):e85266. doi: 10.1371/journal.pone.0085266 (PMC3887030; doi:10.1371/journal.pone.0085266)
Supplement: Supporting Information S1 — A. Glossary of cytogenetic terms used throughout the article. B. Results and discussion concerning the individual datasets used for our study. (DOCX) [file pone.0085266.s001.docx]

**Supporting Information 1**

**Supporting Information 1A.** Glossary of cytogenetic terms used throughout the article (main manuscript or supporting information).

**Agmatoploidy**: Mutations consisting on fissions in holocentric chromosomes that give rise to increasing (continuous or almost) chromosome number series (Malheiros Gardé and Gardé 1950). There is a negligible variation in DNA content.

**Allopolyploidy**: polyploidy (see below) arisen through the fusion of gametes from different species (Rieger et al. 1968).

**Aneuploidy**: Process that gives rise to increasing or decreasing (continuous or almost) chromosome series because of loss or gain of one or a few chromosomes implying important variation in DNA content

**Demipolyploidy**: Event leading to the formation of polyploid series of chromosome numbers in which all numbers arise through the crossing of gametes with different ploidy levels (2*x*, 4*x*, 8*x*, ...). As a result, demipolyploid levels are obtained (4*x*, 6*x*, 10*x*, 12*x* ...) (Mayrose et al. 2010).

**Dysploidy**: Process that gives rise to increasing or decreasing (continuous or almost) chromosome series due to chromosome rearrangements with no important changes in DNA content (Ehrendorfer 1964; Dyer 1979).

**Holocentric or holokinetic chromosome**: Chromosomes with non-localized centromeres (Schrader 1935). The kinetochore is distributed along the whole chromosome (Nagaki et al. 2005).

**Monocentric chromosomes**: Chromosomes with a localized and single centromere. The kinetochore is restricted to the centromeric region (Denburg 2001).

**Polyhaploidy**: Derivation of functional haploid plants from polyploids through haploid partenogenesis (Anderson 1972). It may be understood as a “reversion of polyploids back to diploids” (Stebbins 1970).

**Polyploidy:** chromosome multiplication entailing the addition of complete chromosome sets (3*x*, 4*x*, 5*x*, 6*x*, 7*x*, 8*x*, 9*x*, 10*x*, 11*x*, 12*x* ...) (Otto 2007). In this study we considered ancient and recent polyploidy (paleopolyploidy and neopolyploidy, respectively; see main text).

**Polyploidy *s.s.* (PP) or whole genome duplication (WGD)**: whole chromosome set doubling (2*x*, 4*x*, 8*x*, 16*x*…) (Soltis et al. 2009; Mayrose et al. 2010).

**Symploidy**: Mutations consisting on fusions in holocentric chromosomes that give rise to decreasing (continuous or almost) chromosome number series (Luceño and Guerra 1996). There is a negligible variation in DNA content.

**Supporting Information 1B.** Specific results and discussion concerning the individual datasets used for our study.

***Apiales, Araliaceae: Hedera***. *Hedera* is a north temperate genus with 12 species distributed in the Old World. This genus constitutes a polyploid series with four ploidy levels based on *x =* 24 (*2n* = 2*x*, 4*x*, 6*x*, 8*x*; Vargas et al. 1999), which are highly congruent with species taxonomy (Rutherford et al. 1993; Valcárcel and Vargas 2010). The high chromosome number of the extant diploids in *Hedera* (*2n* = 2*x =* 48) seems to be the result of ancient polyploidy followed by diploidization processes. Coexistence of diploid-like polyploids (*2n* = 2*x*) together with recent polyploids (*2n* = 4*x*, 6*x*, 8*x*) among extant *Hedera* species, leads to infer two different basic chromosome numbers, one primary (*x =* 12), and the other secondary (*x =* 24; Jacobsen 1954; Vargas et al. 1999; Valcárcel et al. 2003). This is also in agreement with the basic chromosome number (*x =* 12) traditionally inferred for the whole family (Goldblatt 1980, 1981; Goldblatt and Johnson 1991; Fedorov 1974), although a recent study (Yi et al. 2004) suggests *x =* 6 as a possible additional basic number.

We investigated the cytogenetic evolution in *Hedera* using the molecular phylogeny from Valcárcel et al. (2003), and the chromosome data from McAllister in Vargas et al. (1999). Our sampling was complete as it included all 12 *Hedera* species together with their chromosome numbers. The phylogeny was dated with the oldest reliable known fossil of *Hedera* (Rim 1994). Our results indicated CRD as the best model of chromosome evolution in *Hedera* (Table 2). Polyploidy (including demipolyploidy) is supported as the main evolutionary mechanism, while gains and losses of single chromosomes are negligible (Table 2). Ancestral state reconstruction, under the best-fitting model, suggests *x =* 24 as the primitive basic number at the root of *Hedera*, with a probability of 0.92 (Table 2). The analysis predicts two polyploidization *s.s.* (PP) and five demipolyploidizations events (demi-PP; Table 2). Particularly, one PP event (from *n* = 24 to *n* = 48) was inferred at the base of the tree for one of the two main lineages (polyploid lineage, *x =* 48), and another one at the origin of *H. colchica* (from *n* = 48 to *n* = 96). Towards the terminal positions within the polyploid lineage, five demi-PP events (from *n* = 48 to *n* = 72) are retrieved.

***Asparagales, Orchidaceae: Subtribe Orchidinae.*** Subtribe Orchidinae (including former Habenariinae subtribe) comprises around 56 genera and 1800 species (Pridgeon et al. 1997; Inda et al. 2010) that can be mostly found in temperate areas of Europe, Africa and Asia. Orchidinae includes some of the most species-rich genera in orchids, and it comprises the majority of orchids growing in Europe and, most particularly, in the Mediterranean Basin (Bateman et al. 2003). Recent dated molecular phylogenies based on nuclear (ITS), mitochondrial (*cox1*) and plastid (*rpl16*) data (Inda et al. 2012) have highlighted the monophyly and the recent (Mid-Oligocene) origin of this subtribe, for which an African or SE Asian origin has been postulated (Bateman et al. 2003). Basic chromosome numbers in subtribe Orchidinae have been established as *x =* 16, 18, 20 and 21 (Pridgeon et al*.* 2001), with *x =* 21 as the ancestral value and *x =* 16 and *x =* 18 representing derived states (D’emerico, 2001; Luo, 2004). Moreover, basic chromosome numbers in the family Orchidaceae are *x =* 6 or *x =* 7 (D’emerico 2001; Greilhuber and Ehrendorfer 1975). Therefore, several rounds of chromosome number increases must have taken place to explain the high chromosome numbers observed in this group.

To analyse the cytogenetic evolution of subtribe Orchidinae we used the dated phylogeny with 103 species published by Inda et al. (2012). Chromosome numbers for 73 species were obtained from different sources including the revision published by D’Emerico et al. (2001) and a public database (IPCN, Missouri Botanical Gardens 2012). Our results highlight CRD as the best model of chromosome evolution in subtribe Orchidinae (Table 2). Under this model, losses of single chromosomes appears as the dominant evolutionary process. Leitch et al. (2009) found correlation between chromosome number and size but no correlation between chromosome number and genome size. They concluded that transitions in chromosome number arise by Robertsonian rearrangements (fission or fusion of chromosomes) at or near the centromere which generate telocentric or metacentric chromosomes. In congruence, when we analyzed available chromosome number and genome size data (http://data.kew.org/cvalues/), we found no correlation (see Supporting Information 2). All of this evidence leads us to conclude that the general pattern for this group is disploidy. Ancestral state reconstruction under the best fitting model indicates *x =* 21 as the basic number at the root of Orchidinae with a probability of 0.88. This analysis also predicts a high number of chromosome losses (32 events) throughout the evolution of the Orchidinae, whereas gains of single chromsomes are far less common (one event). Some events of losses of single chromosomes were reconstructed at the base of some of the most diversified clades within Orchidinae, such as the *Ophrys* + *Serapias* + *Himantoglossum* + *Anacamptis* clade. As regards to PP and demi-PP, the former is more frequent (three vs one event), which is not consistent with the results obtained under the CRDE model. Moreover, inferred PP and demi-PP events affect terminal taxa in some recently diversified clades such as the *Dactylorhiza* and the *Serapias* clades. Our findings are consistent with previous results (Pridgeon et al. 1997). According to these authors, most chromosome number changes in the Orchidinae took place through individual chromosome losses, whereas PP is restricted to a few clades such as the *Dactylorhiza*-*Coeloglossum* clade (Pillon et al. 2007), the *Nigritella*-*Gymnadenia* clade (Hedrén et al. 2000) and the *Habenaria* clade (Luo 2004).

***Asterales, Asteraceae: Bellis, Bellium and Bellidastrum***. *Bellis* (15 species) and *Bellium* (five species) are the only Astereae genera (Asteraceae) endemic to the Mediterranean basin. They are sister groups in a monophyletic clade in turn sister to the monotypic genus *Bellidastrum*, distributed in Europe (Fiz et al. 2002). The first molecular (nrITS) and morphological phylogeny reconstructed an ancestral basic chromosome number of *x =* 9 for this complex (Fiz et al. 2002). All *Bellium* species have the same chromosome number (*n* = 9). In *Bellis*, four polyploid species are present in two main lineages, suggesting multiple origins of polyploidy within the genus (Fiz et al. 2000). Two species seem to be the result of allopolyploidyzation processes (*Bellis* *cordifolia*, *n* = 36, 45; *Bellis* *sylvestris*, *n* = 18, 27). Moreover, the origin of *Bellis* *azorica* (*n* = 36) and *Bellis* *annua* (*n* = 9, 45) remains uncertain since only one ploidy level has been registered. We investigated the cytogenetic evolution in these three genera using the molecular phylogeny and chromosome data from Fiz et al. (2002), and divergence times from Fiz-Palacios and Valcárcel (2011). Our analyses could not discriminate between CRDE and CRD as the model of chromosome evolution that best fit the data, with a slight ∆AIC difference of only 0.04 in favour of CRDE; however, ancestral state reconstruction and inferred events are similar under both models. Under the CRDE model, PP (ρ = 0.0720 events my^-1^) and demi-PP (μ = 0.0225 events my^-1^), with moderately low rates as well as gains of single chromosomes (λ = 0.0089 events my^-1^), with very low rate, are supported while losses of single chromosomes (δ) was equal to zero. Ancestral state reconstruction indicates a primitive basic number of *x =* 9 for the common ancestor of the three genera, with a high probability of 0.99 (Table 2). The same basic number was suggested as the most probable at the origin of *Bellis* (0.99) and *Bellium* (0.99). Six PP events were detected in *Bellis*: two in *Bellis cordifolia* and *B. azorica*, and one event in each of the two *B. sylvestris* samples (which is not monophyletic).

***Asterales, Asteraceae: Helianthus***. This genus is composed of 49 species mainly distributed in North America. In addition to the economic importance of the sunflower (*H. annuus*), this genus has been used as a model for the study of diploid and polyploid hybrid speciation (Time et al. 2007). At least thirteen species of the genus are known to be polyploid. A molecular phylogeny of *Helianthus* based on nrETS sequences revealed that all the main lineages of the genus were embedded within the polyphyletic and perennial sect. *Divaricati* (Timme et al. 2007). These were the biphyletic sect. *Ciliares*, the monophyletic annual sect. *Helianthus*, and the monotypic sect. *Agrestis* (Timme et al. 2007). Three different haploid numbers are known, *n* = 17, 34 and 51, which fit with diploid (*2n* = 34), tetraploid (*2n* = 68) and hexaploid (*2n* = 102) levels. Using the molecular phylogeny and chromosome numbers for 95% of extant species by Timme et al. (2007), the cytogenetic evolution of the genus was analyzed using probabilistic models, of which the best fitting one was CRD (Mayrose et al. 2010). PP and demi-PP at similar rates were suggested as the main agents in the cytogenetic evolution of *Helianthus*, with null rates of single chromosome gains and losses (Mayrose et al. 2010). In our study, we have reanalyzed this dataset with an updated version of the software ChromEvol used by Mayrose et al. (2010), and including a calibration point in the phylogeny (Torices 2010) to yield absolute times (the tree was ultrametric in our analysis in contrast to in Mayrose et al. (2010). CRD was also selected as the best model in our analyses (∆AIC of 2 with CRDE; Table 2). The inferred rate of PP and demi-PP was medium (ρ = 0.2648 events my^-1^), whereas gains and losses of single chromosomes were zero. Our results are also in agreement with those of Mayrose et al. (2010) regarding ancestral reconstruction of the basic number, with *x =* 17 as strongly supported (probability of 0.96: Table 2) at the base of the tree. Eleven events of PP and 11 of demi-PP were inferred by the analyses.

***Brassicales, Resedaceae***. The Resedaceae is a small family composed of six genera and c. 85 species mainly distributed in the temperate regions of the Old World, with a diversity centre in the Mediterranean basin. Known chromosome numbers of the family range from *n* = 6 to *n* = 40. Eigsti (1936) detected a congruent pattern between the different basic numbers and the infrageneric classification of *Reseda*. Later on, a series of cytogenetic studies on the Iberian species of *Reseda* and *Sesamoides* was published, where different basic numbers (*x =* 5, 6, 7) were proposed for the different taxonomic groups (Gonzalez Aguilera et al. 1980a, 1980b; Fernández Peralta and González Aguilera, 1982; González Aguilera and Fernández Peralta 1981, 1983). A scenario of gains and losses of single chromosomes at the origin of the main groups, followed by polyploidization events, was afterwards suggested to explain the cytogenetic evolution of the family (Gonzalez Aguilera and Fernández Peralta 1984). These authors suggested *x =* 5 as the ancestral chromosome number for the family, from which *x =* 6 may have been originated at least twice, whereas a single origin was proposed for *x =* 7. A recent molecular phylogeny of the family based on nuclear and plastid sequences (nrITS, *trnL-F*; Martín-Bravo et al. 2007) revealed that the three main lineages of the phylogeny corresponded to the three tribes traditionally recognised in Resedaceae (Cayluseae, Astrocarpeae and Resedeae). The patterns of cytogenetic evolution inferred from the phylogeny were mostly in agreement with the hypothesis proposed by González Aguilera and Fernández Peralta (1984).

We investigated the cytogenetic evolution in Resedaceae using the nuclear and plastid combined molecular phylogeny and chromosome data for 35 species (36 taxa) from Martín-Bravo et al. (2007). The most frequent chromosome number was selected for the few species which showed intraspecific cytogenetic variation. Genome size data were mainly obtained from González Aguilera and Fernández Peralta (1984), with a few additional values from Hanson et al. (2001) and Suda et al. (2005) (see http://data.kew.org/cvalues/). The phylogenetic tree was ultrametrized with estimations of diversification times taken from Martín-Bravo et al. (2010). Our results indicate CRDE as the best model of chromosome evolution in Resedaceae (Table 2), although closely followed by CRD (∆AIC of only 0.2 between CRDE and CRD). Again, the uncertainty was whether PP and demi-PP occur at the same rates or not. In CRDE model, PP (ρ = 0.087 events my^-1^) and gains of single chromosomes (λ = 0.186 events my^-1^), with moderately low and normal rates, respectively, are supported as the main evolutionary mechanisms (Table 2). Demi-PP rate was comparatively lower (μ = 0.0216 events my^-1^), and losses of single chromosomes (δ) was equal to zero. The inferred rate of constant gains in the alternative CRD model was similar (λ = 0.1742 events my^-1^), whereas polyploidization rate was moderately low (0.0588 events my^-1^). Models which did not include polyploidization (CRND, LRND) displayed largely increased values of AIC and were consequently rejected. Chromosome number of species was not significantly correlated with genome size in clades affected by gains and losses of single chromosomes (see Supporting Information 2), suggesting that gains of single chromosomes do not necessarily entail increases of genome size. Therefore, gains and losses of single chromosomes appear to be mainly dysploid transitions in Resedaceae, contrary to the hypothesis by González Aguilera and Fernández Peralta (1984). Ancestral state reconstruction did not clearly retrieve a primitive basic number at the root of Resedaceae: *x =* 3 and *x =* 4 were the most likely, but both received a rather low probability of 0.25 (Table 2). Our analysis inferred 21 chromosome gains, 10 PP and one demi-PP events. Specifically, the model predicts a gain of one or two chromosomes (from 3 or 4 to 5) at the basal part of the tree, in the branches leading from the root (tribe *Cayluseae*) to tribes Astrocarpeae and Resedeae. An additional independent gain from 5 to 6 could have taken place during the early diversification of one subclade of tribe Resedeae. Other possible gains of single chromosomes are depicted towards terminal branches (*n* = 12 or 13 to *n* = 14 or 15). At least two independent PP events are supported, at the origin of the tribe Astrocarpeae and of the other subclade of tribe Resedaeae. In addition, other PP cannot be discarded in the diversification of *Sesamoides* and tribe Resedeae.

***Caryophyllales, Caryophyllaceae: Arenaria* sect. *Plinthine.*** This section constitutes a monophyletic group (Valcárcel et al. 2006) of 14 morphologically cohesive Mediterranean species (McNeill 1962; López González 1990). The whole genus *Arenaria* is characterised by high cytogenetic variability, with eight different basic chromosome numbers (*x =* 8, 9, 10, 12, 13, 14, 15, 23; Favarger 1962; Contandriopoulos and Favarger 1983). Particularly, section *Plinthine* is highly interesting from a cytogenetic point of view not only because the great range of chromosome numbers detected (from *2n* = 18 to *2n* = 140) but also because the different evolutionary mechanisms likely involved (Nieto Feliner 1985, 2000; Favarger and Nieto Feliner 1988). Although differences in chromosome numbers are scattered within the section, the greatest variability is concentrated in two species. On the one hand, the polyploid series based on *x =* 10 described for *A. tetraquetra* (*n* = 2*x*, 3*x*, 4*x*, 5*x*, 6*x*, 7*x*; Favarger, 1962; Favarger and Nieto Feliner, 1988), and the extensive chromosome series from gains and losses of single chromosomes detected in *A. erinacea* (*n* = 10, 14, 15, 20, 22, 24, 26, 27, 29, 30, 34; Nieto Feliner 2000). Favarger and Nieto Feliner (1988) proposed a scenario in which hybridization, back-crosses and polyploidization may have led to the different ploidy levels of *A. tetraquetra* as a result of glacial and postglacial range shifts. Molecular phylogenetic results (Valcárcel et al. 2006) rejected previous evolutionary hypothesis and revealed the existence of additional underlying evolutionary processes (hybridization, lineage sorting, biased concerted evolution) that greatly difficult the reconstruction of the evolutionary history of sect. *Plinthine*.

We investigated the cytogenetic evolution in *Arenaria* sect. *Plinthine* using the ITS molecular phylogeny from Valcárcel et al. (2006) and chromosome data from Nieto Feliner (1985, 2000) and Favarger and Nieto Feliner (1988). Our sampling was complete as it included all the 14 species of sect. *Plinthine* and chromosome numbers for all of them. Divergence times were estimated using ITS mutation rates (Kay et al. 2006), as implemented in Hipp et al. (2010). Our results indicate LR as the best model of chromosome evolution (Table 2; ∆AIC of 1.4 between LR and LRD). Exceptionally high rates of gains of single chromosomes (λ = 20.1486 events my^-1^) and moderately high rates of losses of single chromosomes (δ = 4.9572 events my^-1^) are supported as the main evolutionary mechanisms, while PP is comparatively lower (ρ = 0.7888 events my^-1^). In addition, the model retrieved high to very high rates of gains and losses of single chromosomes, inversely (λ1 = -1.8536 – -15.987 events my^-1^) and directly (δ1 = 9.3848 – 80.944 events my^-1^) proportional to the number of chromosomes, respectively. Ancestral state reconstruction could not infer any primitive basic number at the root of *Arenaria* sect. *Plinthine* with a probability over 0.05. Due to the poor resolution at deep nodes of the ITS phylogeny, together with the incongruence between taxonomy and molecular data (Valcárcel et al. 2006), caution is required when reconstructing the cytogenetic evolution for this group. An exceptionally high number of chromosome mutation events were inferred: 920 increasing and 1270 losses of single chromosomes, along with 46 PP (Table 2). The exceptionally high number of gains and losses of single chromosomes inferred for this group may be either a methodological issue due to the poor internal resolution of the phylogeny or a biological footprint of the main evolutionary forces involved (Valcárcel et al. 2006). There is a general trend throughout the tree nodes for the expectations of loss events to be higher than the gains. However, we cannot describe a tendency of losses of single chromosomes from the root to the tips since we failed to recover the basic chromosome number for the root of the tree as well as for the next and single internal node. In the same way, the failure to infer the basic chromosome number for the root coupled with the poor internal resolution and the placement of duplication events scattered in internal and external nodes prevented us from setting up the impact of polyploidization. More specifically, we cannot rule out whether gains and losses of single chromosomes events have been preceded by polyploidization shifts or not. Distinguishing between aneuploidy or disploidy remains also open since no information on DNA genome size has ever reported for these plants. However, Nieto Feliner (1985) suggested dysploidy for *A. vittoriana* (*2n* = 30, 32) based on the same size of all chromosomes observed in the counts.

***Geraniales, Geraniaceae: Erodium***. The genus *Erodium* has c. 74 species and it is worldwide distributed, with two main centres of diversity in the western and eastern Mediterranean basin. A first molecular phylogeny based on plastid *trn*L-F sequences (Fiz et al. 2006) identified two main lineages within *Erodium* (clades I and II), which were confirmed by a later study based on nrITS (Fiz et al. 2010). Diversity of chromosome number not only among different species but also within species (e.g. *E. cicutarium* 2*n* = 20, 36, 40, 42, 48, 54, 60) has hampered ancestral chromosome number reconstruction (Fiz et al. 2006). As a result, the ancestral number inferred for the origin of the whole genus from a parsimony reconstruction was equivocal (Fiz et al. 2006). Conversely, three basic chromosome number have been proposed for clade I (*x =* 8, 9, 10), whereas *x =* 10 has been suggested for clade II (Fiz et al. 2006).

We investigated the cytogenetic evolution in *Erodium* using the combined nuclear-plastid (ITS - *trn*L-F) phylogeny and divergence times from Fiz-Palacios et al. (2010) and the chromosome data from Fiz et al. (2006). The data support CRDE as the best model of chromosome evolution in *Erodium* (Table 2). In the CRDE model, moderately low rates of PP (ρ = 0.0455 events my^-1^), gains (λ = 0.0455 events my^-1^), and losses of single chromosomes (δ = 0.0134 events my^-1^) were inferred, whereas the obtained demi-PP rate was negligible (μ = 0.008 events my^-1^). Ancestral state reconstruction supports *x =* 10 at the root of *Erodium* with a probability of 0.45, whereas *x =* 9 received a probability of 0.36 (Table 2). Our analyses suggest a complex pattern of cytogenetic evolution, including nine PP, one demi-PP, two gains and three single chromosome losses. Interestingly, losses of one chromosome (*n* = 10 to *n* = 9) were inferred along the branches leading to nine species of subgen. *Absinthiodea* *pro parte* and to *E. guttatum*. Two single chromosome losses (*n* = 10 to *n* = 8) are suggested at the origin of *E. stephanianum*. These gains and losses of single chromosomes do not appear to be correlated with polyploidy except for the monotypic genus *California* which is sister to *Erodium*. However, since genome size evolution in *Erodium* has not been previously studied it is difficult to differentiate between aneuploidy and dysploidy. Polyploidization is the most important event reconstructed at the *E. botrys* and *E. brachycarpum* clade (from *n* = 10 to *n* = 20), the node leading to *E. heywoodi* and *E. macrocalyx* (from *n* = 10 to *n* = 40) and at the origin of *E. manescavi* (from *n* = 10 to *n* = 20). Our results suggest an interesting pattern of PP (*n* = 10 to *n* = 20) followed by a demi-PP event (*n* = 20 to *n* = 30) in *E. carolinianum*. This may be the outcome of a polyploidization followed by hybridization with the parental species.

***Lamiales, Plantaginaceae: Antirrhineae***. The tribe Antirrhineae includes 29 genera and c. 326 species distributed both in the New and the Old World, with chromosome numbers ranging from *n* = 6 to *n* = 28. The group has been considered as a good example of parallelism between taxonomy and chromosome number (Sutton 1988). While Eurasian genera seemingly constitute a series of gains and losses of single chromosomes between *n* = 6 and *n* = 9, higher chromosome numbers are generally prevalent in the American genera. This scenario could be explained by different hypotheses: (1) the Eurasian chromosome numbers are ancestral, with higher numbers in American genera resulting from polyploidization (Elisens 1985); (2) high chromosome numbers are ancestral, and have yielded lower numbers by losses of single chromosomes (Sutton 1988). Phylogenetic analyses of nuclear (ITS) and plastid (*ndh*F) regions suggest six main lineages within Antirrhineae (Ghebrehiwet et al. 2000; Olmstead et al. 2001; Vargas et al. 2004), all of them containing more than one chromosome number. Variation of chromosome numbers in the three lineages with genera from the New and the Old World has been hypothesized to be the result of multiple, independent events of gains and losses of single chromosomes and polyploidy (Vargas et al. 2004).

For the cytogenetic reconstruction, we employed an extended *ndh*F phylogeny of Antirrhineae including 44 species representing 27 genera (Vargas et al. in prep.). Chromosome numbers were available for 36 species and obtained from Sutton (Sutton 1988). Divergence times were taken from Vargas et al. (in prep.). LRDE is supported as the best model of chromosome evolution. An ancestral chromosome number of *x =* 9 is strongly supported at the root of tribe Antirrhineae, with a probability of 0.96 (Table 2). Increase in chromosome number may have only occurred by a moderately low PP rate (ρ = 0.0117 events my^-1^) or, to a much lesser extent, by demi-PP (μ = 0.0021 events my^-1^). Our results rule out gains of single chromosomes as an important mechanism for this group (λ = 0; λ1 = 0). Decrease in chromosome number may have occurred by losses of single chromosomes with a moderately low rate dependant on the current number of chromosomes (δ = 0; δ1 = 0.0170 – 0.0578 events my^-1^). Available genome size data for Antirrhineae range from 2C = 0.88 to 1.87 pg (Suda et al*.*, 2005; Siljak-Yakovlev et al*.*, 2010; Zaitlin and Pierce 2010; Castro et al*.*, 2012; see http://data.kew.org/cvalues/). For lineages involved in gains and losses of single chromosomes, there seems to be no correlation between genome size (2C) and chromosome number (Supporting Information 2), which suggests a predominance of dysploidy. In addition, several instances of chromosome loss after polyploidy or demipolyploidy are reconstructed by ChromEvol analyses.

Ancestral state reconstruction suggests an intermediate evolutionary scenario between those previously proposed (Elisens 1985; Sutton 1988). A total of 21 chromosome mutations were retrieved by the analysis, of which the majority corresponded to chromosome losses (14 events), followed by PP (six) and a single demi-PP. The highest number (*n* = 9) among Eurasian genera is ancestral, and lower numbers are obtained by recurrent events of losses of single chromosomes. On the other hand, higher numbers in American genera are obtained by independent events of PP or demi-PP, sometimes followed by losses of single chromosomes, as already hypothesized by Vargas et al. (2004). Putative events of recent PP are also inferred in terminal branches of genera *Linaria* and *Kickxia*.

**Malphigiales, Passifloraceae: *Passiflora*.** The tropical genus *Passiflora* L. contains more than 530 species and is widely distributed from southern Argentina into southern United States with an additional 20 species restricted to the Old World. Recent molecular studies have revealed a high congruence between the phylogeny and the chromosome number distribution (Hansen et al. 2006). The two largest lineages in the genus are *Decaloba* and *Passiflora* with typical chromosome numbers of *n* = 6 and *n* = 9, respectively, whereas two smaller subgenera (*Astrophea* and *Deidamioides*) have a haploid number of *n* = 12. The basic chromosome number of the genus is controversial, and several numbers have been proposed (*x =* 6, 9, 10, 12; de Melo and Guerra 2003). Hansen et al. (2006) constructed a Bayesian phylogeny of the genus based on molecular chloroplast data (*rpoC1* intron and the *trnL-T* spacer region) and they reconstructed ancestral chromosome numbers with a MP methodology. The authors hypothesized a chromosome number of *x =* 12 at the base of the genus and losses of single chromosomes with no PP events. Using Hansen et al.’s (2006) dataset and the same probabilistic models as the present study, Mayrose et al. (2010) reexamined chromosome evolution in the genus, inferring that the best models were CRND and CRD with no power to discriminate between them. We have reanalyzed the same dataset but using an updated version of the software chromEvol and adding absolute time estimates from Hearn (2006). We found that the best supported model is CRD (Table 2). The inferred transition rates are very low (λ = 0.0013 events my^-1^, ρ = 0.004 events my^-1^ and δ = 0 events my^-1^). The predicted basic chromosome number at the root of the tree is *x =* 6 with a probability of 0.99, as was already inferred by Mayrose et al. (2010). The inferred mutation events are also mostly congruent with Mayrose et al.’s (2010) results (Table 2). There is not much information about the type of gains and losses of single chromosomes in *Passiflora* (de Melo et al. 2001; de Melo and Guerra 2003). Nevertheless, available genome size data for *Passiflora* (see http://data.kew.org/cvalues/) seem to indicate no correlation between genome size (2C) and chromosome number (Supporting Information 2), which suggests a predominance of dysploidy in this genus.

***Malvales, Cistaceae***. The Cistaceae is a medium-sized family consisting of eight genera and c. 180 species distributed in the Mediterranean region and North America. Known chromosome numbers range from *n* = 9 to *n* = 24, and they are constant in all genera except for *Helianthemum* and *Tuberaria*. Phylogenetic analysis of two plastid regions (*rbc*L, *trn*L-F) in representatives of all eight genera revealed five major lineages (Guzmán and Vargas 2009). A high number (*n* = 16) is found in the basal lineage *Fumana*, while *n* = 9 occurs in the *Cistus*-*Halimium* crown group. Moreover, the haploid chromosome number *n* = 10 is the most common in the *Helianthemum*-*Crocanthemum*-*Hudsonia* lineage (displaying *n* = 5 and *n* = 11 too), whereas the pattern in the *Tuberaria* lineage (*n* = 7, 12, 18, 24) is more complex and no chromosome counts are available for *Lechea tripetala*. Low resolution was found in a parsimony reconstruction of chromosome number in Cistaceae (Guzmán and Vargas 2009).

We analyzed the plastid matrix and cytogenetic information for 53 taxa using times of diversification from Guzmán and Vargas (2009), and found CR as the best supported model of cytogenetic evolution (Table 2). An ancestral chromosome number of *x =* 4 is inferred at the origin of Cistaceae, with a probability of 0.87 (Table 2). Among mutation rates, which are moderately low, PP has the highest (ρ = 0.0434 events my^-1^), followed by gains of chromosomes (λ = 0.0351 events my^-1^), whereas losses of chromosomes was null. Nine PP and seven gains were inferred by the analysis. A wide variation of genome sizes has been reported for the *Halimium*-*Cistus* clade, with *n* = 9 (2C = 3.66 to 7.61 pg; Ellul et al*.*, 2002; Boscaiu et al*.*, 2008). However, genome size data for other Cistaceae are scarce and only include two additional haploid numbers (*n* = 7, 10) (Bennett and Smith 1991; Boscaiu et al*.* 2008). With the available data, there is no correlation between genome size and haploid number (Supporting Information 2), which could suggest a predominance of dysploidy. Additional genome size data are required to confirm this result. According to our reconstruction, the high number (*n* = 16) found in the basal lineage *Fumana* resulted from two consecutive events of PP from *n* = 4. A complement of 5 chromosomes (*n* = 5) appears to be ancestral to the *Helianthemum*-*Crocanthemum*-*Hudsonia* lineage. Given the occurrence of *n* = 5 in a derived species (*H. squamatum*), the common occurrence of *n* = 10 within this lineage is inferred as resulting from recurrent events of PP. Polyhaploidy may have happened in *H. squamatum*, but this mechanism was not included in our modelling as it is a very improbable cytogenetic mechanism (Mayrose et al. 2010). One event of loss of single chromosome may have produced *n* = 11 in *Helianthemum*-*Crocanthemum*-*Hudsonia* lineage. A haploid number *n* = 9 is supported as ancestral to the *Halimium*-*Cistus* crown group, and may have resulted from successive events of PP and gains and losses of single chromosomes from the ancestral basic number *n* = 4. The same number is maintained along the whole lineage.

***Poales, Cyperaceae: Cariceae***. Tribe Cariceae (ca. 2000 spp.; Cyperaceae) is worldwide distributed, mainly in cold and temperate zones of the northern Hemisphere; it is composed of five genera (*Carex*, *Schoenoxiphium*, *Kobresia*, *Uncinia* and *Cymophyllus*). *Carex*, with c. 2000 species in four subgenera (*Carex*, *Vignea*, *Vigneastra* and *Psyllophora*) is one of the richest genera of angiosperms (Reznicek 1990; Egorova 1999). Recent phylogenetic studies (Waterway et al. 2009) indicate that tribe Cariceae is arranged in four main clades: (1) *Carex* sect. *Siderosticta* (subgen. *Carex*), which is sister to the remaining species of the tribe, (2) *Carex* subgen. *Carex* and *Vigneastra*, (3) *Carex* subgen. *Vignea* and (4) the Caricoid clade including *Carex* subgen. *Psyllophora* and representatives of the remaining genera *Schoenoxiphium*, *Uncinia*, *Kobresia* and *Cymophyllus*.

All Cyperaceae species have holocentric chromosomes (without localized centromeres), which allows normal segregation of chromosome fragments during meiosis. As a result, chromosome fission and fusion events are very frequent and have produced a remarkable chromosome number diversification within Cyperaceae (e.g. genus *Carex*, *n* = 6 to *n* = 62; Roalson, 2008; Hipp et al. 2013). In contrast, polyploidization events are very rare in *Carex* (Hipp et al., 2013). There are several studies which demonstrate the absence of direct relation between chromosome number transitions and genome size changes in *Carex* (Roalson, 2008; Chung et al., 2011, 2012; Lipnerová et al., 2013).

We simulated the chromosome evolution in tribe Cariceae using the molecular phylogeny with 100 species (nrDNA ITS and ETS-1f; plastid *trn*L and *trn*L-F) published by Waterway et al. (2009) and re-analysed in Escudero et al. (2012), the synopsis of chromosome numbers reported in Roalson (2008) and the diversification times published by Escudero et al. (2012). Our results indicate the best model of chromosome evolution is LR. The LR model results show that rates of chromosome gains and losses are high either for a single chromosome (λ = 3.3090 events my^-1^ and δ =1.9570 events my^-1^) or for a single chromosome proportional to the chromosome number (λ1 = 0.6162 – 6.8986 events my^-1^ and δ1 = 0.3107 – 3.4798 events my^-1^). Conversely, rates of PP are very low (ρ = 0.007 my^-1^). In addition, the results from the different alternative models display uncertainty about whether there is demi-PP or not and its rates. Ancestral state reconstruction does not suggest a clear ancestral chromosome number at the root of the tree. The model does not predict any general pattern of increasing or decreasing of chromosome number (3480 gains and 3699 losses inferred for the whole tree; Table 2). Finally, the reconstruction predicts only three PP events (Table 2).

***Poales, Cyperaceae: Carex* sect. *Ovales*.** *Carex* section *Ovales* (ca. 90 spp. worldwide) is the most species-rich section of the genus *Carex* in the New World. Mayrose et al. (2010), using the nrDNA phylogeny (57 spp., ITS and ETS sequences) and chromosome data presented in Hipp (2007), inferred that the best model of chromosome evolution was LRND and that chromosome evolution had proceeded from higher to lower numbers. In the present study, we have reanalyzed this dataset with an updated version of ChromEvol and absolute times implemented in the branch length (age estimations from Escudero et al. 2010). Our results are congruent with those in Mayrose et al. (2010) as the best model retrieved is also LRND (Table 2). The main cytogenetic processes within this group are gain (λ = 23.1041 events my^-1^) and loss (δ = 0.1386 events my^-1^) of a single chromosome and gain (λ1 = 0.5675 – 0.9534 events my^-1^) and loss (δ1 = 16.828 – 28.270 events my^-1^) of a single chromosome proportional to the current chromosome number. While the inferred single gain and linear loss rates were very high, single loss and linear gain ones were medium. PP and demi-PP events were not supported (Table 2). Ancestral state reconstruction could not infer a basic chromosome number with high probability, but suggested an evolutionary decreasing of chromosome number, since probability of greater chromosome numbers at the base of the tree was higher. This was already proposed by Mayrose et al. (2010) and Hipp (2007).

***Poales, Cyperaceae: Carex* sect. *Phacocystis***. It is one of the largest section of the genus, constituted by c. 70 species mainly distributed in cold and temperate areas of the northern Hemisphere (Dragon and Barrington 2009). It is arranged in four major clades, characterized by a geographical rather than taxonomical adscription (Dragon and Barrington 2008). Reported chromosome numbers range from *n* = 27 to *n* = 48 (Standley et al. 2002). Agmatoploidy and symploidy processes appear to be common in the section and are probably responsible for the short chromosomal series found within several taxa (Faulkner 1972; Cayouette and Morisset 1985; Luceño and Aedo 1994). However, no general pattern of increasing or decreasing dysploidy has been proposed for the group. Remarkably, sect. *Phacocystis* is one of the few groups within Cyperaceae for which rare instances of polyploidization have been reported, in the form of intraspecific triploids (Faulkner 1972).

Our approach to the cytogenetic evolution of sect. *Phacocystis* uses the molecular phylogeny (nrDNA ITS and ETS, plastid *psb*A-*trn*H) and divergence times from Dragon and Barrington (2009) and the chromosome data from Roalson (2008), for a total of 21 species (24 taxa). The results indicate CRND as the best model in sect. *Phacocystis* (Table 2). The main evolutionary processes inferred within this group are gains (λ = 1.1723 events my^-1^) and losses (δ = 1.5425 events my^-1^) of a single chromosome, both with moderately high rates. Neither PP nor demi-PP events were retrieved (Table 2). Like in *Carex* sect. *Spyrostachyae* (see below), more chromosome losses than gains were inferred by the analysis (101 and 74 events, respectively). Ancestral state reconstruction suggests *x =* 38 and *x =* 37 as the ancestral chromosome number, but with a low probability of 0.32 and 0.23, respectively (Table 2). The model predicts a gain of two chromosomes at the origin of the Eurasian clade, represented by *C. nigra* and *C. elata* (*n* = 38 to *n* = 40). Consecutive gain processes were detected in two clades: (1) *C. lenticularis* clade (*n* = 38 to *n* = 43) and (2) *C. aquatilis* clade (*n* = 38 to *n* = 39).

***Poales, Cyperaceae: Carex* sect. *Spirostachyae***. This section is formed by c. 40 species (c. 75 including sect. *Echinochlaenae* with ca. 35), mainly distributed in the Mediterranean and tropical-subtropical regions around the world. It is formed by two main clades named subsects. *Spirostachyae* and *Elatae* (Escudero and Luceño 2009). Chromosome numbers range almost continuously from *n* = 30 to *n* = 42. Increasing of chromosome number has been thought to be prevalent over decreasing processes and *n* = 30 has been proposed as the ancestral number for the section (Luceño and Castroviejo 1993). Polyploidization events have never been detected in sect. *Spirostachyae*. In congruence, Escudero et al. (2010) found a continuous pattern of chromosome evolution without any shift in chromosome number.

We performed an approach to the cytogenetic evolution in sect. *Spirostachyae* using the molecular phylogeny of 38 species (nrDNA ITS, plastid 5´*trn*K intron) from Escudero et al. (2009) and chromosome data and divergence times from Escudero et al. (2010). Our results indicate CRND as the best model of chromosome evolution in *Spirostachyae* (Table 2). Moderate rates of gains (λ = events 0.1644 my^-1^) and losses (δ = events 0.3855 my^-1^) of a single chromosome were supported as the single evolutionary processes. The model does not predict any general pattern of increasing or decreasing of chromosomes, but losses (34 events inferred) rather than gains (12 events) seem to be more frequent (Table 2). PP or demi-PP were discarded (Table 2). Ancestral state reconstruction suggests *x =* 38 (P = 0.27) or *x =* 39 (P = 0.24) as the ancestral chromosome number at the root of the tree, but with rather low probabilities (Table 2). In subsect. *Spirostachyae* (*n* = 35), the model predicts losses of chromosomes at the origin of *C. extensa* group (*n* = 35 to *n* = 32), and a single gain for the oriental lineage of *C. distans* group (*n* = 35 to *n* = 36). In subsect. *Elatae,* four main events are supported: (1) a loss of a single chromosome in the *C. perraudieriana* clade (*n* = 38 to *n* = 37), (2) four consecutive single losses in the *C. punctata* group (*n* = 38 to *n* = 34), (3) three losses in the tropical African group (*n* = 38 to *n* = 35) and (4) a loss in *C. laevigata* group (*n* = 38 to *n* = 37).

***Saxifragales, Saxifragaceae: Saxifraga sect. Saxifraga***. *Saxifraga* sect. *Saxifraga* consists of c. 70 species centred on Europe. The genus displays marked karyological instability, as shown by the wide range of chromosome numbers (from *n* = 10 to *n* > 100; Vargas and Nieto Feliner 1995; Webb and Gornall 1989) and the common occurrence of many different haploid numbers within a single species. Furthermore, there is uncertainty about some published counts, given the small size of the chromosomes and the presence of supernumerary chromosomes (Vargas and Nieto Feliner 1995; Webb and Gornall 1989). A basic number *x =* 8 has been suggested for *Saxifraga*, with both polyploid (auto- and allopolyploidy) and gains and losses of single chromosomes likely involved in the origin of other numbers (Webb and Gornall 1989). A nrDNA (ITS sequences) phylogeny of this group (Vargas 2000) yielded highly incongruent relationships between phylogeny and morphological diagnostic characters. Active processes of gains and losses of single chromosomes and allopolyploidy were suggested to play an important role in the evolution of the group, but no reconstruction of chromosome number evolution was implemented (Vargas 2000).

Here we analyzed the ITS matrix from Vargas (Vargas 2000) with additional sequences from Vargas (2001), diversification times estimated from Kay et al. (2006) and chromosome data from Vargas and Nieto Feliner (1995) and Webb and Gornall (1989) for 50 taxa. As expected, the cytological instability and uncertainty about chromosome counts and their relative frequencies led to a high uncertainty in our results. CRD is the best supported model (Table 2). This model supports the occurrence of gains, losses as well as PP and demi-PP with the same rate. There is also high uncertainty about the ancestral basic number at the root of *Saxifraga* sect. *Saxifraga* (Table 2), as ten different basic numbers, from *x =* 20 to *x =* 29, received a probability higher than 0.05. The rates of gains (λ = 1.0956 events my^-1^) and losses (δ = 1.0521 events my^-1^) of single chromosomes were moderately high. In contrast, rates of PP and demi-PP were very low (ρ = 0.0013 my^-1^). There is only one publication on *Saxifraga* DNA content, providing results of one species (*S. granulata*) of the same subgenus (*Saxifraga*), but a different taxonomic section than those herein studied (Redondo et al*.* 1996). *S. granulata* rendered three chromosome numbers (2*n* = 22, 44, 52) and three DNA amounts (2C = 1.35, 3.54, 4.76) related to polyploidy. Nevertheless, eight species (*S. genesiana*, *S. fragilis*, *S. geranioides*, *S. pentadactylis*, *S. intricata*, *S. pubescens*, *S. vayredana*, *S. moschata*) of our study group yielded chromosome numbers (Vargas and Nieto Feliner 1995) uncorrelated with their DNA genome sizes (Supporting Information 2; Mas et al*.*, unpublished). Although there is great chromosome diversity in this group, the results lead us to suggest predominant dysploidy. Reconstruction of chromosome mutations under this scenario of cytological instability (216 losses and 29 PP events inferred) is highly uncertain (data not shown). However, the overall patterns found are congruent with previous assumptions about the importance of dysploidy and polyploidy in the evolution of the group (Vargas 2000).

**References**

Anderson LC (1972) *Flaveria campestris* (Asteraceae): A case of polyhaploidy or relic ancestral diploidy. Evolution 26: 671-673.

Bateman RM, Hollingsworth PM, Preston J, Yi-Bo L, Pridgeon AM, Chase MW (2003) Molecular phylogenetics and evolution of Orchidinae and selected Habenariinae (Orchidaceae). Bot J Linn Soc 142: 1-40.

Bennett MD, Smith JB (1991) Nuclear DNA amounts in angiosperms. Philos Trans R Soc Lond B Biol Sci 334: 309-345.

Boscaiu M, Ellul P, Soriano P, Vicente O (2008) Nuclear DNA content variation in *Halimium* and *Xolantha* (Cistaceae). Plant Biosyst 142: 17-23.

Castro M, Castro S, Loureiro J (2012) Genome size variation and incidence of polyploidy in Scrophulariaceae sensu lato from the Iberian Peninsula. AoB Plants 2012: pls037.

Cayouette J, Morisset P (1986) Chromosome studies on *Carex paleacea* Wahl., *C. nigra* (L.) Reichard, and *C. aquatilis* Wahl. in northeastern North America. Cytologia 51: 857–883.

Chung K-S, Hipp AL, Roalson EH (2012) Chromosome number evolves independently of genome size in a clade with nonlocalized centromeres (*Carex*: Cyperaceae). Evolution 66: 2708-2722.

Chung K-S, Weber JA, Hipp AL (2011) Dynamics of chromosome number and genome size variation in a cytogenetically variable sedge (*Carex scoparia* var. *scoparia*, Cyperaceae). Am J Bot 98: 122-129.

Contandriopoulos J, Favarger C (1983) Sur quelques espéces de Turquie du genre *Arenaria* L. (étude cytotaxonomique). Candollea 38: 733–743.de Melo NF, Guerra M (2003) Variability of the 5S and 45S rDNA sites in *Passiflora* L. species with distinct base chromosome numbers. Ann Bot 92: 309-316.

de Melo NF, Cervi AC, Guerra M (2001) Karyology and cytotaxonomy of the genus *Passiflora* L. (Passifloraceae). Plant Syst Evol 226: 69-84.

D'Emerico S. 2001. Tribe Orchideae cytogenetics. In: Pridgeon AM, Cribb PJ, Chase MW, Rasmussen FN editors. Genera Orchidacearum. Vol. 2, Orchidoideae, part 1. Oxford: Oxford University Press, 216–224.

Dragon JA, Barrington DS (2008) East vs. West: Monophyletic clades within the paraphyletic *Carex acuta* complex, section *Phacocystis* (Cyperaceae). In: Naczi RFC, Ford BA, editors. Sedges: uses, diversity, and systematics of the Cyperaceae. St. Louis: Missouri Botanical Garden Press. pp. 215-226.

Dragon JA, Barrington DS (2009) Systematics of the *Carex aquatilis* and *C. lenticularis* lineages: geographically and ecologically divergent sister clades of *Carex* section *Phacocystis* (Cyperaceae). Am J Bot 96: 1896-1906.

Dyer AF (1979) Investigating chromosomes. London: Edward Arnold. 138 p.

Egorova TV (1999) The sedges (*Carex* L.) of Russia and adjacent states (within the limits of the former USSR). St. Petersburg and St. Louis: St. Petersburg State Chemical-Pharmaceutical Academy and Missouri Botanical Garden. 772 p.

Ehrendorfer F (1964) Cytologie, taxonomie und evolution bei samenpflanzen. Vistas Bot 4: 99-186.

Eigsti OJ (1936) Cytological studies in the Resedaceae. Bot Gaz 98: 363-369.

Elisens WJ (1985) Monograph of the Maurandyinae (Plantaginaceae-Antirrhineae). Syst Bot Monogr 5: 1-97.

Ellul P, Boscaiu M, Vicente O, Moreno V, Rosselló JA (2002) Intra- and interspecific variation in DNA content in *Cistus* (Cistaceae). Ann Bot 90: 345-351.

Escudero M, Hipp AL, Luceño M (2010) Karyotype stability and predictors of chromosome number variation in sedges: A study in *Carex* section *Spirostachyae* (Cyperaceae). Mol Phylogenet Evol 57: 353-363.

Escudero M, Hipp AL, Waterway M, Valente LM (2012) Diversification rates and chromosome evolution in the most diverse angiosperm genus of the temperate zone (*Carex*, Cyperaceae). Mol Phylogenet Evol 63: 650-655.

Escudero M, Luceño M (2009) Systematics and evolution of *Carex* sects. *Spirostachyae* and *Elatae* (Cyperaceae). Plant Syst Evol 279: 163-189.

Escudero M, Valcárcel V, Vargas P, Luceño M (2009) Significance of ecological vicariance and long-distance dispersal in the diversification of *Carex* sect. *Spirostachyae* (Cyperaceae). Am J Bot 96: 2100-2114.

Faulkner JS (1972) Chromosome studies on *Carex* section *Acutae* in north-west Europe. Bot J Linnean Soc 65: 271-300.

Favarger C (1962) L’évolution parallèle du caryotype. Rev Cytol Biol Vég Bot 25: 277-286.

Favarger C, Nieto Feliner G (1988) On the races of *Arenaria* *tetraquetra* (Caryophyllaceae). Bot J Linn Soc 97: 1-8.

Fernández Peralta AM, González Aguilera JJ (1982) Cytogenetic and evolutionary studies on the Spanish species of *Reseda* L.: section *Luteola* Dumort. (Resedaceae). Taxon 31: 1–8.

Fiz O, Valcárcel V, Vargas P (2002) Phylogenetic position of Mediterranean Astereae and character evolution of daisies (*Bellis*, Asteraceae) inferred from nrDNA ITS sequences. Mol Phylogenet Evol 25: 157–171.

Fiz O, Vargas P, Alarcón ML, Aldasoro JJ (2006) Phylogenetic relationships and evolution in *Erodium* (Geraniaceae) based on *trn*L-*trn*F sequences. Syst Bot 31: 739–763.

Fiz-Palacios O, Vargas P, Vila R, Papadopulos A, Aldasoro JJ (2010) The uneven phylogeny and biogeography of *Erodium* (Geraniaceae): radiations in the Mediterranean and recent recurrent intercontinental colonization. Ann Bot 106: 871–884.

Fiz-Palacios O, Valcárcel V (2011) Imbalanced diversification of two Mediterranean sister genera (*Bellis* and *Bellium*, Asteraceae) within the same time frame. Plant Syst Evol 295: 109–118.

Ghebrehiwet M, Bremer B, Thulin M (2000) Phylogeny of the tribe Antirrhineae (Plantaginaceae) based on morphological and *ndh*F sequence data. Plant Syst Evol 220: 223–239.

Goldblatt P (1980) Polyploidy in angisosperms. In: Lewis WH, editor. Polyploidy: Biological Relevance. New York: Plenum Press. pp. 219–239.

Goldblatt P (1981) Index to plant chromosome numbers 1975-1985. Monogr Syst Bot Miss Bot Gard 5: 8.

Goldblatt P, Johnson DE (1991) Index to plant chromosome numbers 1988-1989. Monogr Syst Bot Miss Bot Gard 40: 1–238.

González Aguilera JJ, Fernández Peralta AM (1981) Caryology and evolution in *Sesamoides* (Resedaceae). Plant Syst Evol 139: 147-154.

González Aguilera JJ, Fernández Peralta AM (1983) The nature of polyploidy in *Reseda* sect. *Leucoreseda* (Resedaceae). Plant Syst Evol 142: 223–237.

González Aguilera JJ, Fernández Peralta AM (1984) Phylogenetic relationships in the family Resedaceae. *Genetica* 64: 185-197.

González Aguilera JJ, Fernández Peralta AM, Sañudo A (1980a) Estudios citogenéticos y evolutivos en especies españolas de la familia Resedaceae L. sección *Glaucoreseda* DC. Anales Inst Bot Cavanilles 36: 311–320.

González Aguilera JJ, Fernández Peralta AM, Sañudo A (1980b) Cytogenetic and evolutive studies on the Spanish species of the family Resedaceae L.: sections *Phyteuma* L. and *Resedastrum* Duby. Bol Soc Brot 53: 519–536.

Greilhuber J, Ehrendorfer F (1975) Chromosome numbers and evolution in *Ophrys* (Orchidaceae). Plant Syst Evol 124: 125-138.

Guzmán B, Vargas P (2009) Historical biogeography and character evolution of Cistaceae (Malvales) based on analysis of plastid *rbc*L and *trn*L-*trn*F sequences. Org Div Evol 9: 83-99.

Hansen AK, Gilbert LE, Simpson BB, Downie SR, Cervi AC, Jansen RK (2006) Phylogenetic relationships and chromosome number evolution in *Passiflora*. Syst Bot 31: 138-150.

Hanson L, McMahon KA, Johnson MAT, Bennett MD (2001) First nuclear DNA C-values for 25 angiosperms families. Ann Bot 87: 251-258.

Hearn DJ (2006) *Adenia* (Passifloraceae) and its adaptive radiation: phylogeny and growth form diversification. Syst Bot 31: 805-821.

Hedrén M, Klein E, Teppner H (2000) Evolution of polyploids in the European orchid genus *Nigritella*: evidence from allozyme data. Phyton 40: 239-275.

Hipp AL (2007). Nonuniform processes of chromosome evolution in sedges (*Carex*: Cyperaceae). Evolution 61: 2175-2194.

Hipp AL, Chung KS, Escudero M. In press. Holocentric chromosomes. In: Maloy S, Hughes K, editors. Encyclopedia of Genetics, 2^nd^ Edn. London and New York: Elsevier. pp. 000-000.

Hipp AL, Rothrock PE, Roalson EH (2009) The evolution of chromosome arrangements in *Carex* (Cyperaceae). Bot Rev 75: 96-109.

Hipp AL, Rothrock PE, Whitkus R, Weber JA (2010) Chromosomes tell half of the story: The correlation between karyotype rearrangements and genetic diversity in sedges, a group with holocentric chromosomes. Mol Ecol 19: 3124-3138.

Inda LA, Pimentel M, Chase MW (2010) Contribution of mitochondrial *cox*1 intron sequences to the phylogenetics of tribe Orchideae (Orchidaceae): Do the distribution and sequence of this intron in orchids also tell us something about its evolution? Taxon 59: 1053-1064.

Inda LA, Pimentel M, Chase MW (2012) Phylogenetics of tribe Orchideae (Orchidaceae; Orchidoideae) based on combined DNA matrices: inferences regarding timing of diversification and evolution of pollination syndromes. Ann Bot 110: 71-90.

Jacobsen P (1954) Chromosome numbers in the genus *Hedera* L. Hereditas 40: 252-254.

Kay KM, Whittall JB, Hodges SA (2006) A survey of nuclear ribosomal internal transcribed spacer substitution rates across angiosperms: An approximate molecular clock with life history effects. BMC Evol Biol. 6: 36.

Leich IJ, Kahandawala I, Suda J, Hanson L, Ingrouille MJ, et al. (2009) Genome size diversity in orquids: consequences and evolution. Ann Bot 104: 469-481.

Lipnerová I, Bureš P, Horová L, Šmarda P (2013) Evolution of genome size in *Carex* (Cyperaceae) in relation to chromosome number and genomic base composition. Ann Bot 111: 79-94.

López González G (1990) *Arenaria* (Caryophyllaceae). In: Castroviejo S, editor. Flora Iberica, vol. 2. Madrid: Servicio de Publicaciones del CSIC. pp. 172-224.

Luceño M, Aedo C (1994) Taxonomic revision of the Iberian species of *Carex* L. section *Phacocystis* Dumort. (Cyperaceae). Bot J Linn Soc 114: 183-214.

Luceño M, Castroviejo S (1993) Cytotaxonomic studies in the sections *Spirostachyae* (Drejer) Bailey and *Ceratocystis* Dumort. of the genus *Carex* L. (Cyperaceae) with special reference to Iberian and North African taxa. Bot J Linnean Soc 112: 335-350.

Luceño M, Guerra M (1996) Numerical variations in species exhibiting holocentric chromosomes: a nomenclatural proposal. III-IV. Caryologia 49: 301-309.

Luo YB (2004) Cytological Studies on some representative species of the tribe Orchideae (Orchidaceae) from China. Bot J Linnean Soc 145: 231-238.

Malheiros Gardé N, Gardé A (1950) Agmatoploidia no gênero *Luzula* DC. Genet Iberica 3: 155-176.

Martín-Bravo S, Meimberg H, Luceño M, Märkl W, Valcárcel V, et al. (2007) Molecular systematics and biogeography of Resedaceae based on ITS and *trn*L-F sequences. Mol Phylogenet Evol 44: 1105-1120.

Martín-Bravo S, Valcárcel V, Vargas P, Luceño M (2010) Geographical speciation related to Pleistocene range shifts in the western Mediterranean mountains (*Reseda* sect. *Glaucoreseda*, Resedaceae). Taxon 59: 466-482.

Mayrose I, Barker, MS, Otto SP (2010) Probabilistic models of chromosome number evolution and the inference of polyploidy. Syst Biol 59: 132-144.

McNeill J (1962) Taxonomic studies in the Alsinoideae: I. Generic and infrageneric groups. Notes Royal Bot Gard Edinburgh 24: 79-155.

Nagaki K, Kashihara K, Murata M (2005) Visualization of Diffuse Centromeres with Centromere-Specific Histone H3 in the Holocentric Plant Luzula nivea. Plant Cell 17: 1886-1893

Nieto Feliner G (1985) Datos citotaxonómicos sobre *Arenaria* sect. *Plinthine* (Reichenb.) McNeill. Candollea 40: 471-483.

Nieto Feliner G (2000) Números cromosomáticos de plantas occidentales, 849-854. An Jard Bot Mad 58: 165-166.

Olmstead RG, dePamphilis CW, Wolfe AD, Young ND, Elisons WJ, Reeves PA (2001) Disintegration of the Plantaginaceae. Am J Bot 88: 348-361.

Otto SP (2007) The evolutionary consequences of polyploidy*.* Cell 131: 452-462.

Pillon Y, Fay MF, Hedren M, Bateman RM, Devey DS, et al. (2007) Evolution and temporal diversification of western European polyploid species complexes in *Dactylorhiza* (Orchidaceae). Taxon 56: 1185-1208.

Pridgeon AM, Bateman RM, Cox AV, Hapeman JR, Chase MW (1997) Phylogenetics of subtribe Orchidinae (Orchidoideae, Orchidaceae) based on nuclear ITS sequences. 1. Intergeneric relationships and polyphyly of *Orchis* *sensu lato*. Lindleyana 12: 89-109.

Pridgeon AM, Cribb PJ, Chase MC, Rasmussen FN (2001) Genera Orchidacearum 2: *Orchidoideae*. Oxford: Oxford University Press. 464 p.

Redondo N, Horjales M, Brown S, Villaverde C (1996) Biometric and cytometric study of nuclear DNA within *Saxifraga granulata* L. Bol Soc Brot 67: 287-301.

Reznicek AA (1990) Evolution in sedges (*Carex*, Cyperaceae). Can J Bot 68: 1409-1432.

Rieger R, Michaelis A, Green MM (1968) A glossary of genetics and cytogenetics: Classical and molecular. Berlin: Springer-Verlag. 652 p.

Rim KH (1994) Fossils of North Korea. Pyongyang: Science and Technology Press.

Roalson EH (2008) A synopsis of chromosome number variation in the Cyperaceae. Bot Rev 74: 209-393.

Rutherford A, McAllister HA, Mill RR (1993) New ivies from the Mediterranean area and Macaronesia. The Plantsman 15: 115-128.

Schrader F (1935) Notes on the mitotic behavior of long chromosomes. Cytologia 6: 422-430.

Siljak-Yakovlev S, Pustahija F, Solic EM, Bogunic F, Muratovic E, et al. (2010) Towards a genome size and chromosome number database of Balkan flora: C-values in 343 taxa with novel values for 242. Adv Sci Lett 3: 190-213.

Soltis DE, Albert VA, Leebens-Mack J, Bell CD, Paterson AH, et al. (2009) Polyploidy and angiosperm diversification*.* Am J Bot 96: 336-348.

Standley LA, Cayouette J, Bruederle L (2002) *Carex* sect. *Phacocystis* (Cyperaceae). In: Flora of North America Editorial Committee, editors. Flora of North America, north of Mexico, vol. 23. New York: Oxford University Press. pp. 379–401.

Stebbins GL (1970) Chromosomal evolution in higher plants. Menlo Park: Addison-Wesley Publ. Co. 216 p.

Suda J, Kyncl T, Jarolimova V (2005) Genome size variation in Macaronesian angiosperms: forty percent of the Canarian endemic flora completed. Plant Syst Evol 252: 215-238.

Sutton D (1988) A revision of the tribe Antirrhineae. Oxford: Oxford University Press. 584 p.

Timme RE, Simpson BB, Linder CR (2007) High-resolution phylogeny for *Helianthus* (Asteraceae) using the 18S-26S ribosomal DNA external transcribed spacer. Am J Bot 94: 1837-1852.

Torices R (2010) Adding time-calibrated branch lengths to the Asteraceae supertree. J Syst Evol 48: 271-278.

Valcárcel V, Fiz O, Vargas P (2003) Chloroplast and nuclear evidence for multiple origins of polyploids and diploids of *Hedera* (Araliaceae) in the Mediterranean basin. Mol Phylogenet Evol 27: 1-20.

Valcárcel V, Vargas P, Nieto Feliner G (2006) Phylogenetic and phylogeographic analysis of the W Mediterranean *Arenaria* section *Plinthine* (Caryophyllaceae) based on nuclear, plastid, and morphological markers. Taxon 55: 297-312.

Vargas P (2000) A phylogenetic study of *Saxifraga* sect. *Saxifraga* (Saxifragaceae) based on nrDNA ITS sequences. Plant Syst Evol 223: 59-70.

Vargas P (2001) Phylogenetic and evolutionary insights into the *Saxifraga pentadactylis* complex (*Saxifragaceae*): variation in nrITS sequences. Nordic J Bot 21: 75-82.

Vargas P, Nieto Feliner G (1995) Cytotaxonomical study of *Saxifraga* series *Ceratophyllae* s.l. (*Saxifragaceae*). Plant Syst Evol 197: 209-223.

Vargas P, McAllister HA, Morton C, Jury SL, Wilkinson MJ (1999) Polyploid speciation in *Hedera* (Araliaceae): Phylogenetic and biogeographic insights based on chromosome counts and ITS sequences. Plant Syst Evol 219: 165-179.

Vargas P, Rosselló JA, Oyama R, Güemes J (2004) Molecular evidence for naturalness of genera in the tribe Antirrhineae (Plantaginaceae) and three independent evolutionary lineages from the New World and the Old. Plant Syst Evol 249: 151-172.

Waterway MJ, Hoshino T, Masaki T (2009) Phylogeny, species richness, and ecological specialization in Cyperaceae tribe Cariceae. Bot Rev 75: 138-159.

Webb DA, Gornall RJ (1989) Saxifrages of Europe. London: Christopher Helm. 337 p.

Yi T, Lowry PP, Plunkett GM, Wen J (2004) Chromosomal evolution in Araliaceae and close relatives. Taxon 53: 987-100.

Zaitlin D, Pierce AJ (2010) Nuclear DNA content in *Sinningia* (Gesneriaceae); intraspecific genome size variation and genome characterization in *S. speciosa*. Genome 53: 1066-1082.
