# Supplementary material for: Karyotypic Changes through Dysploidy Persist Longer over Evolutionary Time than Polyploid Changes
Source: PLoS One. 2014 Jan 9;9(1):e85266. doi: 10.1371/journal.pone.0085266 (PMC3887030; doi:10.1371/journal.pone.0085266)
Supplement: Supporting Information S2 — Table S1. Parameters of the best supported model inferred for each group with ChromEvol. Table S2. Species, diploid chromosome number (2n) and genome size (2C, pg) for Cistaceae. Table S3. Species, diploid chromosome number (2n) and genome size (2C, pg) for Antirrhineae. Table S4. Species, diploid chromosome number (2n) and genome size (2C, pg) for Saxifraga sect. Saxigraga. Table S5. Species, diploid chromosome number (2n) and genome size (2C, pg) for Orchidinae. Table S6. Species, diploid chromosome number (2n) and genome size (2C, pg) for Resedaceae. Table S7. Species, diploid chromosome number (2n) and genome size (2C, pg) for Passiflora. Figure S1. Linear model for the correlation between diploid chromosome number (2n) and genome size (2C, pg) in Cistaceae. Figure S2. Linear model for the correlation between diploid chromosome number (2n) and genome size (2C, pg) in Antirrhineae. Figure S3. Linear model for the correlation between diploid chromosome number (2n) and genome size (2C, pg) in Saxifraga sect. Saxigraga. Figure S4. Linear model for the correlation between diploid chromosome number (2n) and genome size (2C, pg) in Orchidinae. Figure S5. Linear model for the correlation between diploid chromosome number (2n) and genome size (2C, pg) in Resedaceae. Figure S6. Linear model for the correlation between diploid chromosome number (2n) and genome size (2C, pg) in Passiflora. (DOCX) [file pone.0085266.s002.docx]

**Table S1**. Parameters of the best supported model inferred for each group with ChromEvol. λ = gains, δ = losses, ρ = polyploidy (or polyploidy and demipolyploidy in CRD model), μ = demipoliploidy, λ1 = gains and δ1 = losses (in λ1 and δ1 rates depend linearly on the current chromosome number). my= million years. CRD = Constant_Rate_Demi, CRDE = Constant_Rate_Demi_Est, LR = Linear_Rate, and LRDE = Linear_Rate_Demi_Est.

|  | Apiales | Asparagales | Asterales | | Brassicales | Caryophyllales | Geraniales | Lamiales |
| --- | --- | --- | --- | --- | --- | --- | --- | --- |
|  | Araliaceae: *Hedera* | Orchidaceae: Orchidinae | Asteraceae: *Bellis*, *Bellium*, *Bellidastrum* | Asteraceae: *Helianthus* | Resedaceae | Caryophyllaceae: *Arenaria* | Geraniaceae: *Erodium* | Scrophulariaceae: Antirrhineae |
| Model | CRD | CRD | CRDE | CRD | CRDE | LR | CRDE | LRDE |
| λ (my^-1^) | 0 | 0.0064 | 0 | 0 | 0.186 | 20.1486 | 0.0134 | 0 |
| δ (my^-1^) | 0 | 0.0838 | 0 | 0 | 0 | 4.9572 | 0.0158 | 0 |
| ρ (my^-1^) | 0.0191 | 0.0039 | 0.0720 | 0.2648 | 0.087 | 0.7888 | 0.0455 | 0.0117 |
| μ (my^-1^) | - | - | 0.0225 | - | 0.0216 | - | 0.0080 | 0.0021 |
| λ1 (my^-1^) | - | - | - | - | - | -0.2317 (-1.8536 – -15.987) | - | 0 |
| δ1 (my^-1^) | - | - | - | - | - | 1.1731 (9.3848 – 80.9440) | - | 0.0034 (0.0170 – 0.0578) |

**Table S1**. Continued. CR = Constant_Rate, LRND = Linear_Rate_No_Dupli, and CRND = Constant_Rate_No_Dupli.

|  | Malphigiales | Malvales | Poales | | | | Saxifragales |
| --- | --- | --- | --- | --- | --- | --- | --- |
|  | Passifloraceae: *Passiflora* | Cistaceae | Cyperaceae: Cariceae | Cyperaceae: *Carex* sect. *Ovales* | Cyperaceae: *Carex* sect. *Phacocystis* | Cyperaceae: *Carex* sect. *Spirostachyae* | Saxifragaceae: *Saxifraga* sect. *Saxifraga* |
| Model | CRD | CR | LR | LRND | CRND | CRND | CRD |
| λ (my^-1^) | 0.0013 | 0.0351 | 3.3090 | 23.1041 | 1.1723 | 0.1644 | 1.0956 |
| δ (my^-1^) | 0 | 0 | 1.9570 | 0.1386 | 1.5425 | 0.3855 | 1.0521 |
| ρ (my^-1^) | 0.0040 | 0.0434 | 0.0070 | - | - | - | 0.0013 |
| μ (my^-1^) | - | - | - | - | - | - | - |
| λ1 (my^-1^) | - | - | 0.1232 (0.6162 – 6.8986) | 0.0227 (0.5675 – 0.9534) | - | - |  |
| δ1 (my^-1^) | - | - | 0.0621 (0.3107 – 3.4798) | 0.6731 (16.828 – 28.270) | - | - |  |

**Table S2**. Species, diploid chromosome number (*2n*) and genome size (2C, pg) for Cistaceae.

| ***Species*** | **Diploid number (*2n*)** | **Genome size (2C, pg)** |
| --- | --- | --- |
| *Cistus osbeckiifolius* | 18 | 4.13 |
| *Cistus symphytifolius* | 18 | 4.91 |
| *Cistus albidus* | 18 | 4.78 |
| *Cistus crispus* | 18 | 3.92 |
| *Cistus creticus* | 18 | 4.34 |
| *Cistus heterophyllus* | 18 | 4.82 |
| *Cistus parviflorus* | 18 | 4.96 |
| *Cistus populifolius* | 18 | 4.29 |
| *Cistus psilosepalus* | 18 | 5.22 |
| *Cistus salviifolius* | 18 | 4.76 |
| *Cistus albanicus* | 18 | 5.34 |
| *Cistus monspeliensis* | 18 | 5.88 |
| *Cistus ladanifer* | 18 | 4.45 |
| *Cistus laurifolius* | 18 | 4.46 |
| *Cistus clusii* | 18 | 5.28 |
| *Cistus libanotis* | 18 | 5.77 |
| *Helianthemum nummularium* | 20 | 4.5 |
| *Halimium calycinum* | 18 | 7.61 |
| *Halimium atriplicifolium* | 18 | 3.66 |
| *Xolantha tuberaria* | 14 | 3.34 |

**Figure S1**. Linear model for the correlation between diploid chromosome number (*2n*) and genome size (2C, pg) in Cistaceae.

**Table S3**. Species, diploid chromosome number (*2n*) and genome size (2C, pg) for Antirrhineae.

| **Species** | **Diploid number (*2n*)** | **Genome size (2C, pg)** |
| --- | --- | --- |
| *Anarrhinum bellidifolium* | 18 | 1.13 |
| *Anarrhinum durimimium* | 18 | 1.11 |
| *Anarrhinum longipedicellatum* | 18 | 1.12 |
| *Antirrhinum cirrhigerum* | 18 | 1.21 |
| *Antirrhinum linkianum* | 18 | 1.23 |
| *Antirrhinum meonanthum* | 18 | 1.20 |
| *Antirrhimum majus* | 18 | 1.29 |
| *Chaenorhinum origanifolium* | 14 | 1.13 |
| *Cymbalaria muralis* | 14 | 0.99 |
| *Kickxia spuria* | 18 | 1.64 |
| *Kickxia scoparia* | 18 | 1.87 |
| *Linaria aeruginea* | 12 | 1.29 |
| *Linaria amethystea* | 12 | 1.05 |
| *Linaria diffusa* | 12 | 1.15 |
| *Linaria incarnata* | 12 | 1.13 |
| *Linaria polygalifolia* | 12 | 1.32 |
| *Linaria saxatilis* | 12 | 1.21 |
| *Linaria spartea* | 12 | 1.11 |
| *Linaria supina* | 12 | 1.30 |
| *Linaria vulgaris* | 12 | 1.73 |
| *Misopates calycinum* | 16 | 0.88 |
| *Misopates orontium* | 16 | 0.88 |

**Figure S2**. Linear model for the correlation between diploid chromosome number (*2n*) and genome size (2C, pg) in Antirrhineae.

**Table S4**. Species, diploid chromosome number (*2n*) and genome size (2C, pg) for *Saxifraga* sect. *Saxigraga*.

| **Species** | **Diploid number (*2n*)** | **Genome size (2C, pg)** |
| --- | --- | --- |
| *Saxifraga genesiana* | 44 | 1.75 |
| *Saxifraga fragilis* | 64 | 1.74 |
| *Saxifraga geranioides* | 52 | 1.7 |
| *Saxifraga pentadactylis* | 32 | 1.49 |
| *Saxifraga intricata* | 33 | 1.43 |
| *Saxifraga pubescens* | 27 | 1.42 |
| *Saxifraga vayredana* | 63 | 1.36 |
| *Saxifraga moschata* | 28 | 1.31 |
| *Saxifraga granulata* | 22 | 1.35 |
| *Saxifraga granulata* | 44 | 3.54 |
| *Saxifraga granulata* | 52 | 4.76 |

**Figure S3**. Linear model for the correlation between diploid chromosome number (*2n*) and genome size (2C, pg) in *Saxifraga* sect. *Saxigraga*.

**Table S5**. Species, diploid chromosome number (*2n*) and genome size (2C, pg) for *Orchidinae*.

| **Species** | **Diploid number (*2n*)** | **Genome size (2C, pg)** |
| --- | --- | --- |
| *Anacamptis pyramidalis* | 36 | 24.64 |
| *Dactylorhiza fuchsii* | 40 | 5.78 |
| *Dactylorhiza incarnata pulchella* | 40 | 7.09 |
| *Dactylorhiza sambucina* | 40 | 14 |
| *Gymnadenia conopsea* | 40 | 11.01 |
| *Herminium monorchis* | 40 | 14.8 |
| *Himanthoglossum robertianum* | 36 | 18.32 |
| *Platanthera bifolia* | 42 | 13.74 |
| *Platanthera chlorantha* | 42 | 22.12 |
| *Traunsteinera globosa* | 42 | 12.38 |

**Figure S4**. Linear model for the correlation between diploid chromosome number (*2n*) and genome size (2C, pg) in *Orchidinae*.

**Table S6**. Species, diploid chromosome number (*2n*) and genome size (2C, pg) for Resedaceae.

| **Species** | **Diploid number (*2n*)** | **Genome size (2C, pg)** |
| --- | --- | --- |
| *Reseda complicata* | 28 | 1.71 |
| *Reseda glauca* | 28 | 2.11 |
| *Reseda gredensis* | 28 | 2.63 |
| *Reseda virgata* | 28 | 1.44 |
| *Reseda luteola* | 26 | 1.02 |
| *Reseda luteola* | 24 | 1.75 |
| *Reseda alba* | 40 | 1.45 |
| *Reseda barrelieri* | 20 | 1.68 |
| *Reseda suffruticosa* | 20 | 0.92 |
| *Reseda undata* | 20 | 1.22 |
| *Reseda paui* | 20 | 1.33 |
| *Reseda scoparia* | 30 | 0.70 |
| *Reseda lanceolata* | 24 | 1.7 |
| *Reseda lutea* | 48 | 1.37 |
| *Reseda stricta* | 24 | 2.86 |
| *Reseda media* | 12 | 2.09 |
| *Reseda phyteuma* | 24 | 1.34 |
| *Sesamoides purpurascens* | 20 | 1.32 |
| *Sesamoides suffruticosa* | 60 | 1.02 |

**Figure S5**. Linear model for the correlation between diploid chromosome number (*2n*) and genome size (2C, pg) in Resedaceae.

**Table S7**. Species, diploid chromosome number (*2n*) and genome size (2C, pg) for *Passiflora*.

| ***Species*** | **Diploid number (*2n*)** | **Genome size (2C, pg)** |
| --- | --- | --- |
| *Passiflora suberosa* | 24 | 0.92 |
| *Passiflora foetida* | 20 | 1.39 |
| *Passiflora marifloia* | 12 | 1.39 |
| *Passiflora miersii* | 18 | 1.4 |
| *Passiflora cincinnata* | 18 | 1.42 |
| *Passiflora caerulea* | 18 | 1.58 |
| *Passiflora edulis* | 18 | 1.58 |
| *Passiflora coccinea* | 18 | 1.62 |
| *Passiflora amethystina* | 18 | 1.68 |
| *Passiflora mucronata* | 18 | 1.7 |
| *Passiflora edmundoi* | 18 | 1.72 |
| *Passiflora galbana* | 18 | 1.76 |
| *Passiflora pentagona* | 24 | 1.84 |
| *Passiflora serrato-digitata* | 18 | 1.86 |
| *Passiflora maliformis* | 18 | 1.89 |
| *Passiflora laurifolia* | 18 | 1.94 |
| *Passiflora giberti* | 18 | 1.96 |
| *Passiflora menispermifolia* |  | 2.28 |
| *Passiflora nitida* | 18 | 2.41 |
| *Passiflora quadrangularis* | 18 | 2.68 |

**Figure S6**. Linear model for the correlation between diploid chromosome number (*2n*) and genome size (2C, pg) in *Passiflora*.
