# Supplementary material for: Karyotypic Changes through Dysploidy Persist Longer over Evolutionary Time than Polyploid Changes
Source: PLoS One. 2014 Jan 9;9(1):e85266. doi: 10.1371/journal.pone.0085266 (PMC3887030; doi:10.1371/journal.pone.0085266)

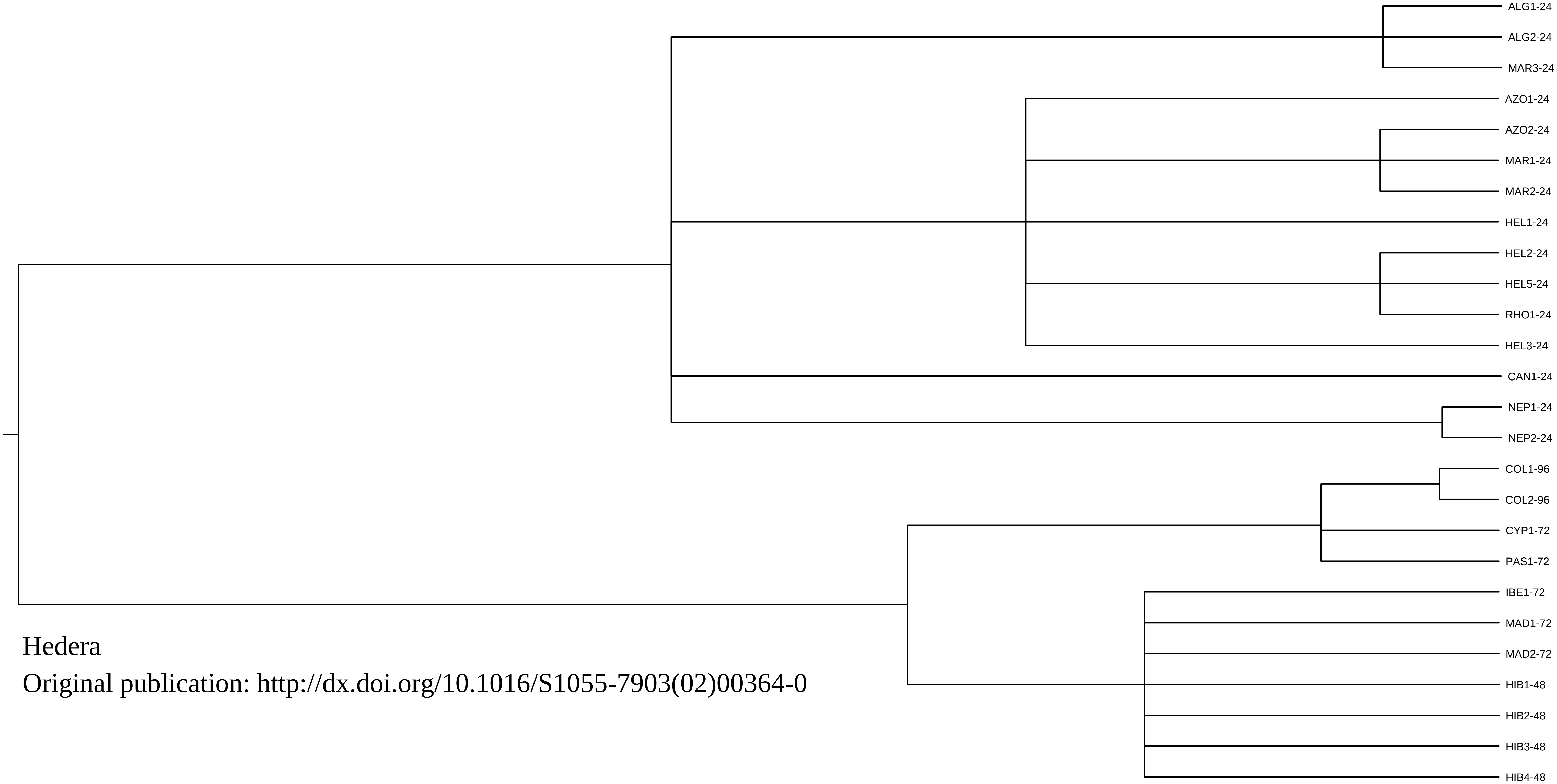

Hedera

Original publication: http://dx.doi.org/10.1016/S1055-7903(02)00364-0

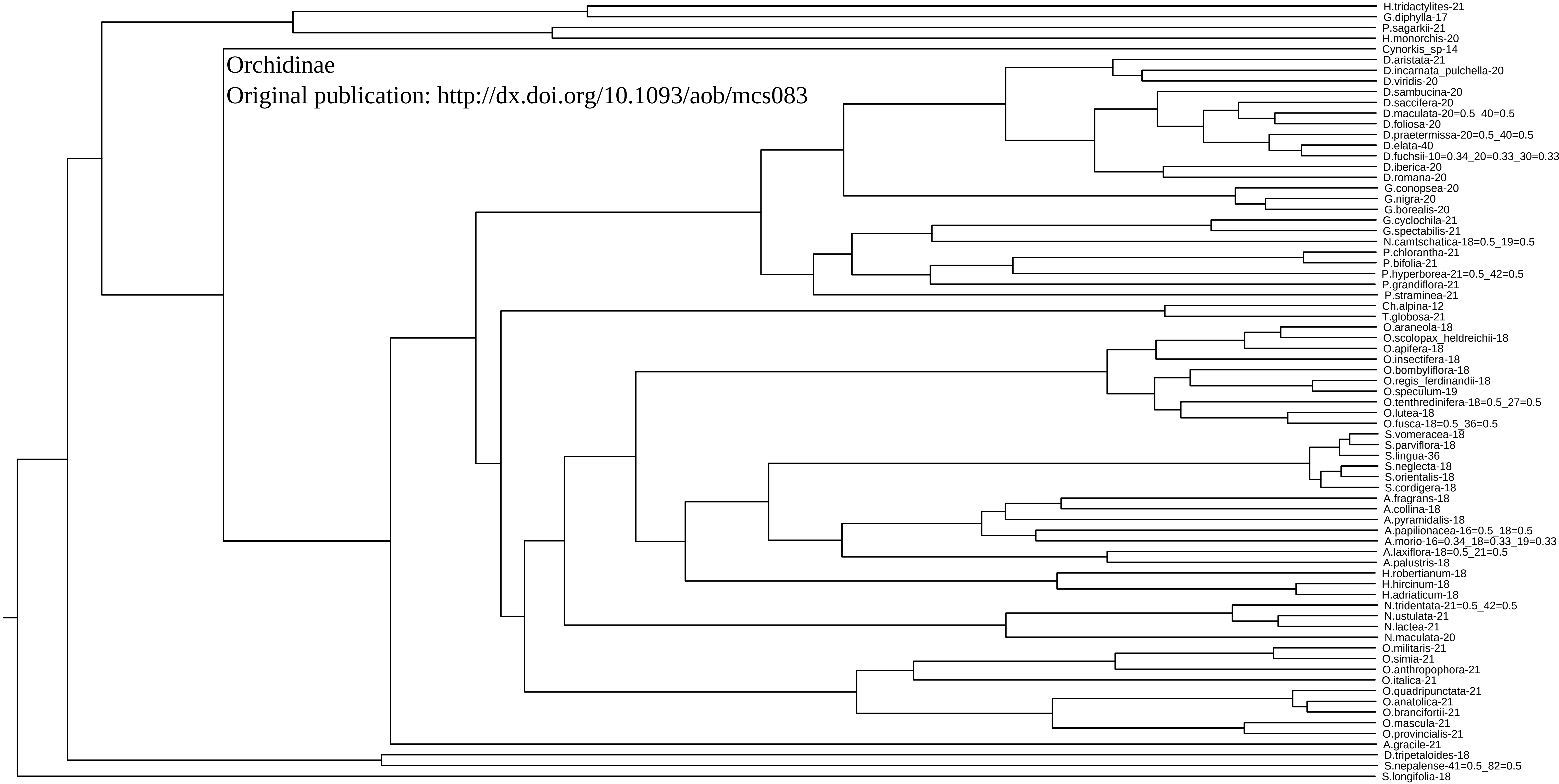
Orchidinae
Original publication: http://dx.doi.org/10.1093/aob/mcs083
H.tridactylites-21
G.diphylla-17
P.sagarkii-21
H.monorchis-20
Cynorkis_sp-14
D.aristata-21
D.incarnata_pulchella-20
D.viridis-20
D.sambucina-20
D.saccifera-20
D.maculata-20=0.5_40=0.5
D.foliosa-20
D.praetermissa-20=0.5_40=0.5
D.elata-40
D.fuchsii-10=0.34_20=0.33_30=0.33
D.iberica-20
D.romana-20
G.conopsea-20
G.nigra-20
G.borealis-20
G.cyclochila-21
G.spectabilis-21
N.camtschatica-18=0.5_19=0.5
P.chlorantha-21
P.bifolia-21
P.hyperborea-21=0.5_42=0.5
P.grandiflora-21
P.straminea-21
Ch.alpina-12
T.globosa-21
O.araneola-18
O.scolopax_heldreichii-18
O.apifera-18
O.insectifera-18
O.bombyliflora-18
O.regis_ferdinandii-18
O.speculum-19
O.tenthredinifera-18=0.5_27=0.5
O.lutea-18
O.fusca-18=0.5_36=0.5
S.vomeracea-18
S.parviflora-18
S.lingua-36
S.neglecta-18
S.orientalis-18
S.cordigera-18
A.fragrans-18
A.collina-18
A.pyramidalis-18
A.papilionacea-16=0.5_18=0.5
A.morio-16=0.34_18=0.33_19=0.33
A.laxiflora-18=0.5_21=0.5
A.palustris-18
H.robertianum-18
H.hircinum-18
H.adriaticum-18
N.tridentata-21=0.5_42=0.5
N.ustulata-21
N.lactea-21
N.maculata-20
O.militaris-21
O.simia-21
O.anthropophora-21
O.italica-21
O.quadripunctata-21
O.anatolica-21
O.brancifortii-21
O.mascula-21
O.provincialis-21
A.gracile-21
D.tripetaloides-18
S.nepalense-41=0.5_82=0.5
S.longifolia-18

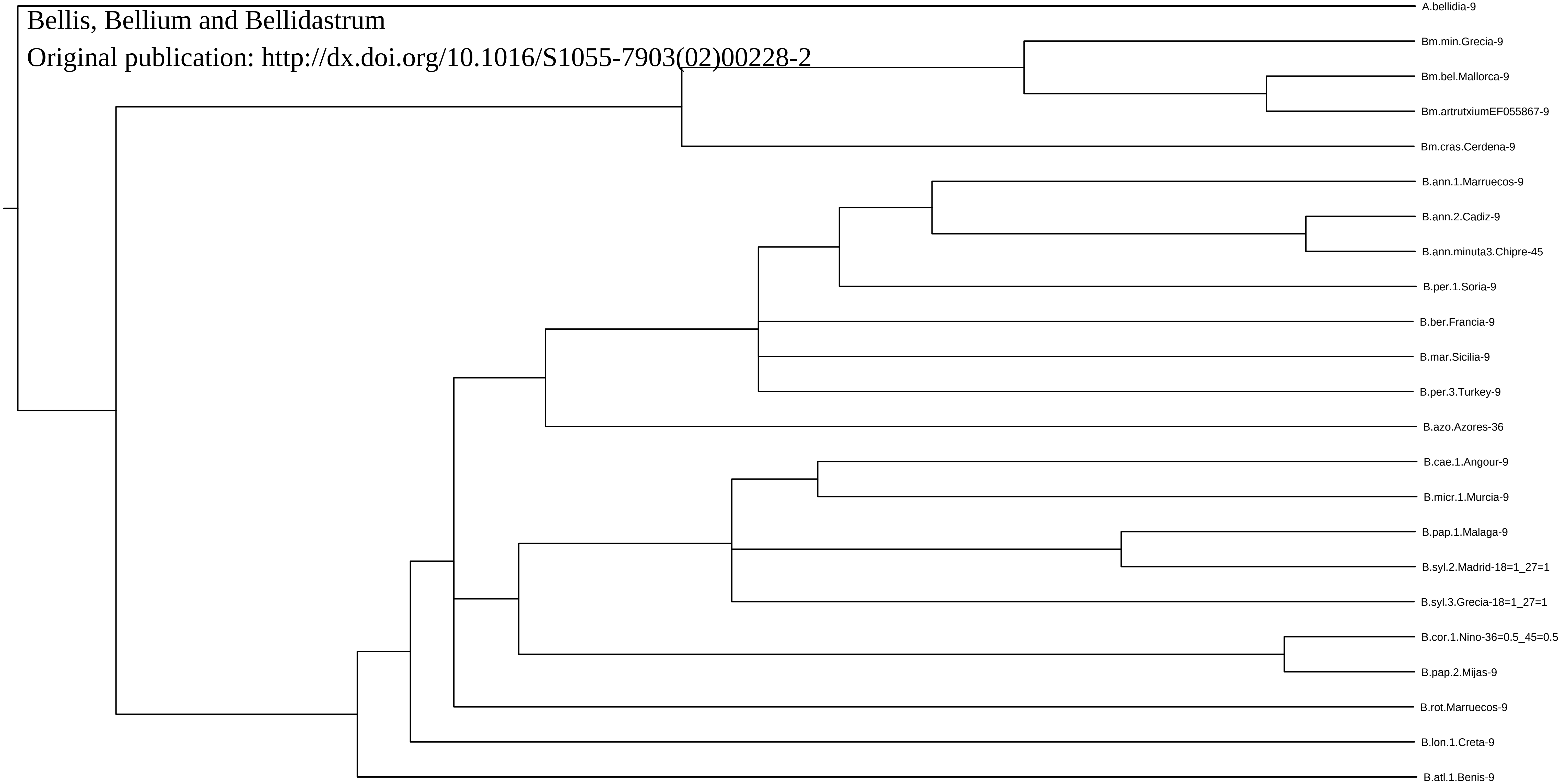
Bellis, Bellium and Bellidastrum
Original publication: http://dx.doi.org/10.1016/S1055-7903(02)00228-2
A.bellidia-9
Bm.min.Grecia-9
Bm.bel.Mallorca-9
Bm.artrutxiumEF055867-9
Bm.cras.Cerdena-9
B.ann.1.Marruecos-9
B.ann.2.Cadiz-9
B.ann.minuta3.Chipre-45
B.per.1.Soria-9
B.ber.Francia-9
B.mar.Sicilia-9
B.per.3.Turkey-9
B.azo.Azores-36
B.cae.1.Angour-9
B.micr.1.Murcia-9
B.pap.1.Malaga-9
B.syl.2.Madrid-18=1_27=1
B.syl.3.Grecia-18=1_27=1
B.cor.1.Nino-36=0.5_45=0.5
B.pap.2.Mijas-9
B.rot.Marruecos-9
B.lon.1.Creta-9
B.atl.1.Benis-9

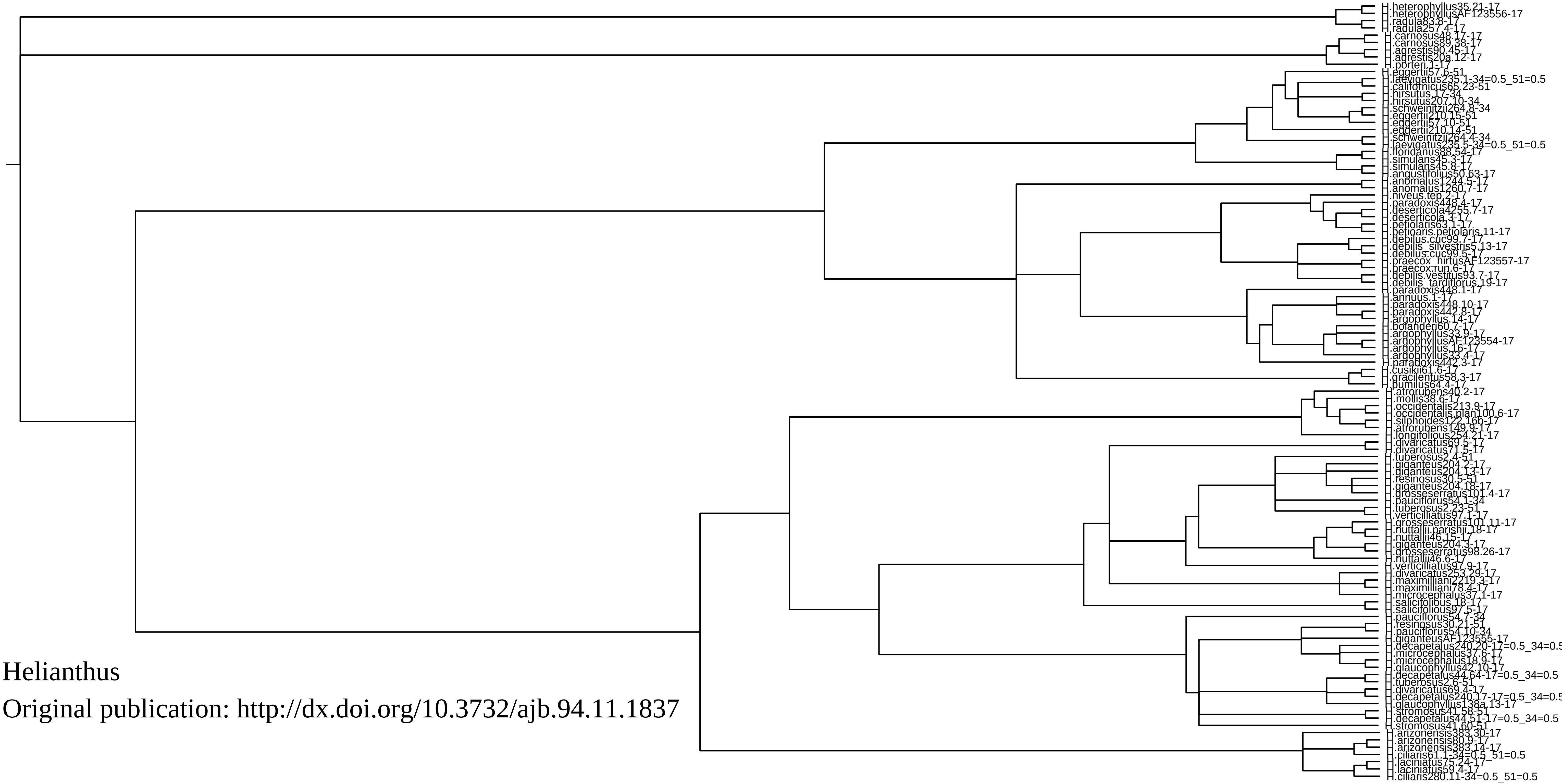

Helianthus

Original publication: http://dx.doi.org/10.3732/ajb.94.11.1837

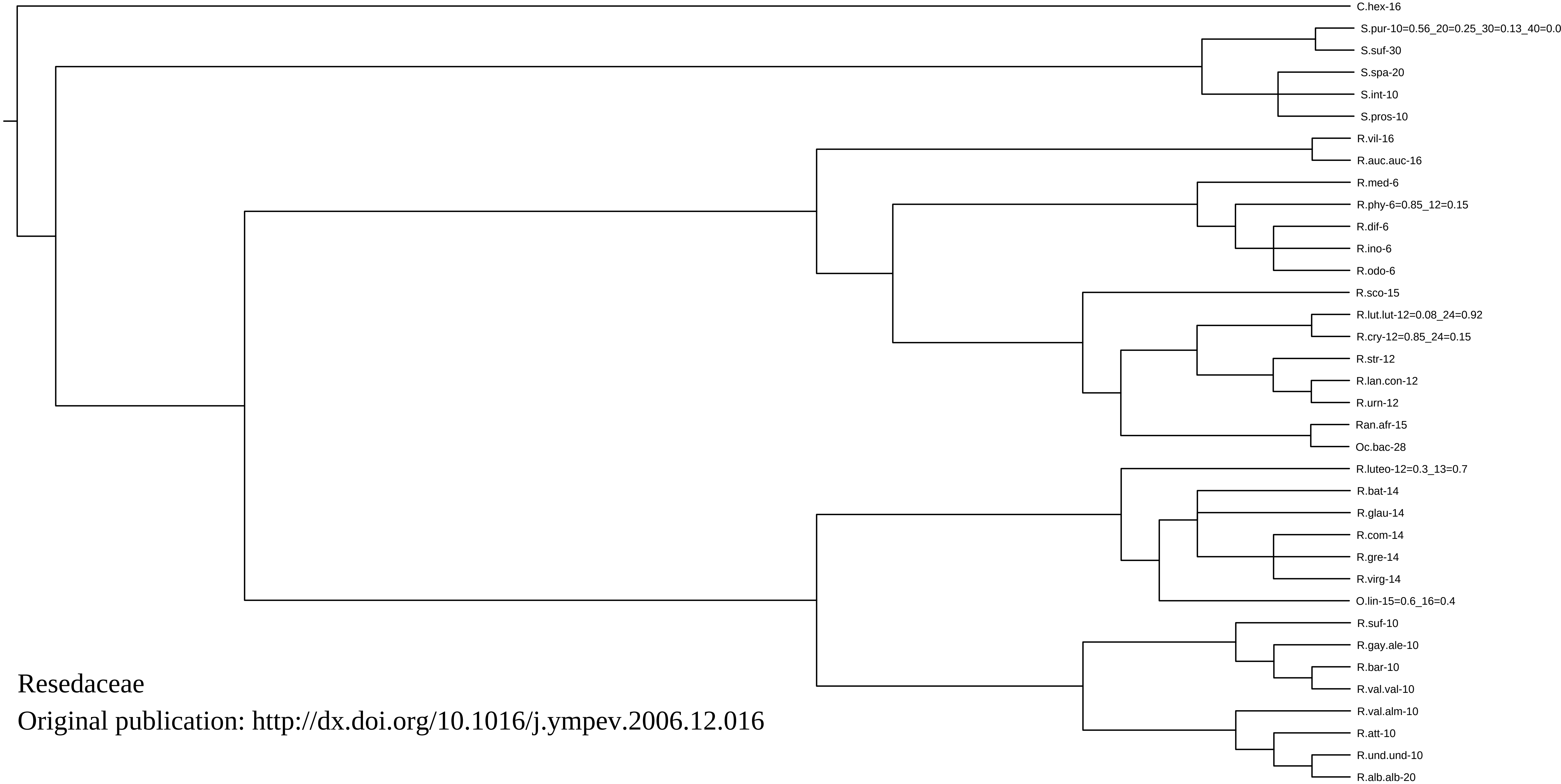

Resedaceae

Original publication: http://dx.doi.org/10.1016/j.ympev.2006.12.016

Arenaria

Original publication: http://www.jstor.org/stable/25065579

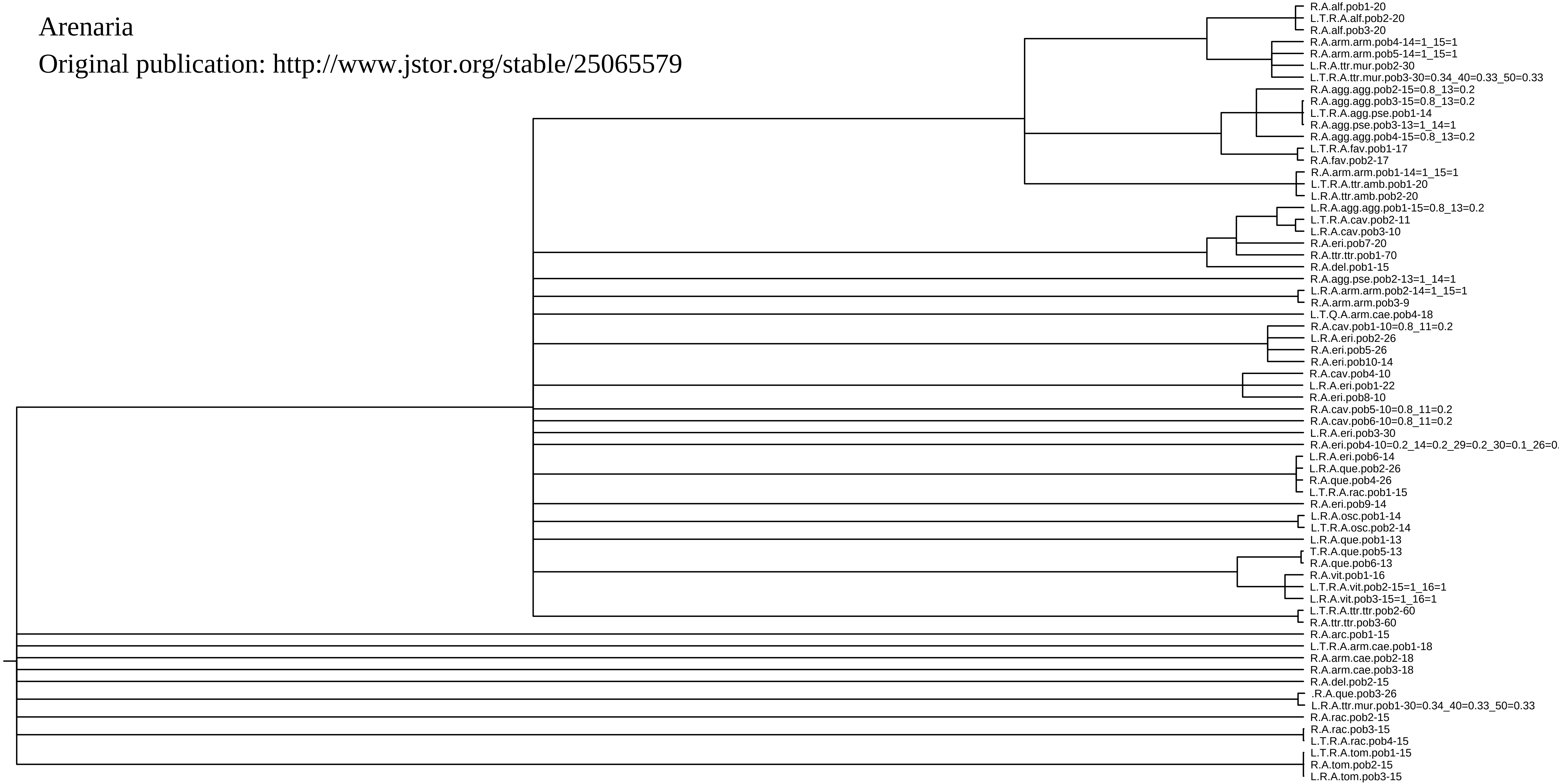

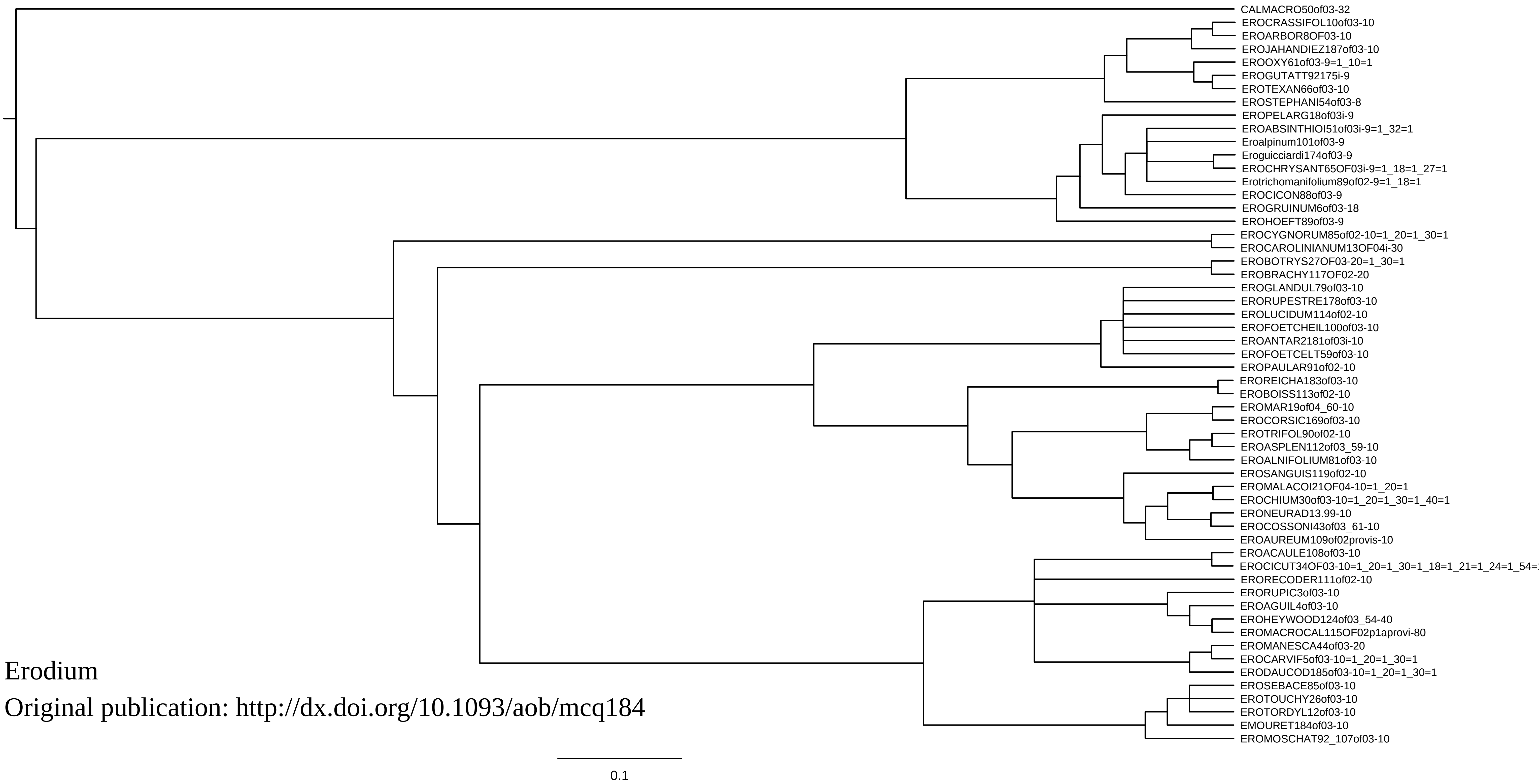

Erodium

Original publication: http://dx.doi.org/10.1093/aob/mcq184

Antirrhineae

Original publication: Vargas et al. (in prep.)

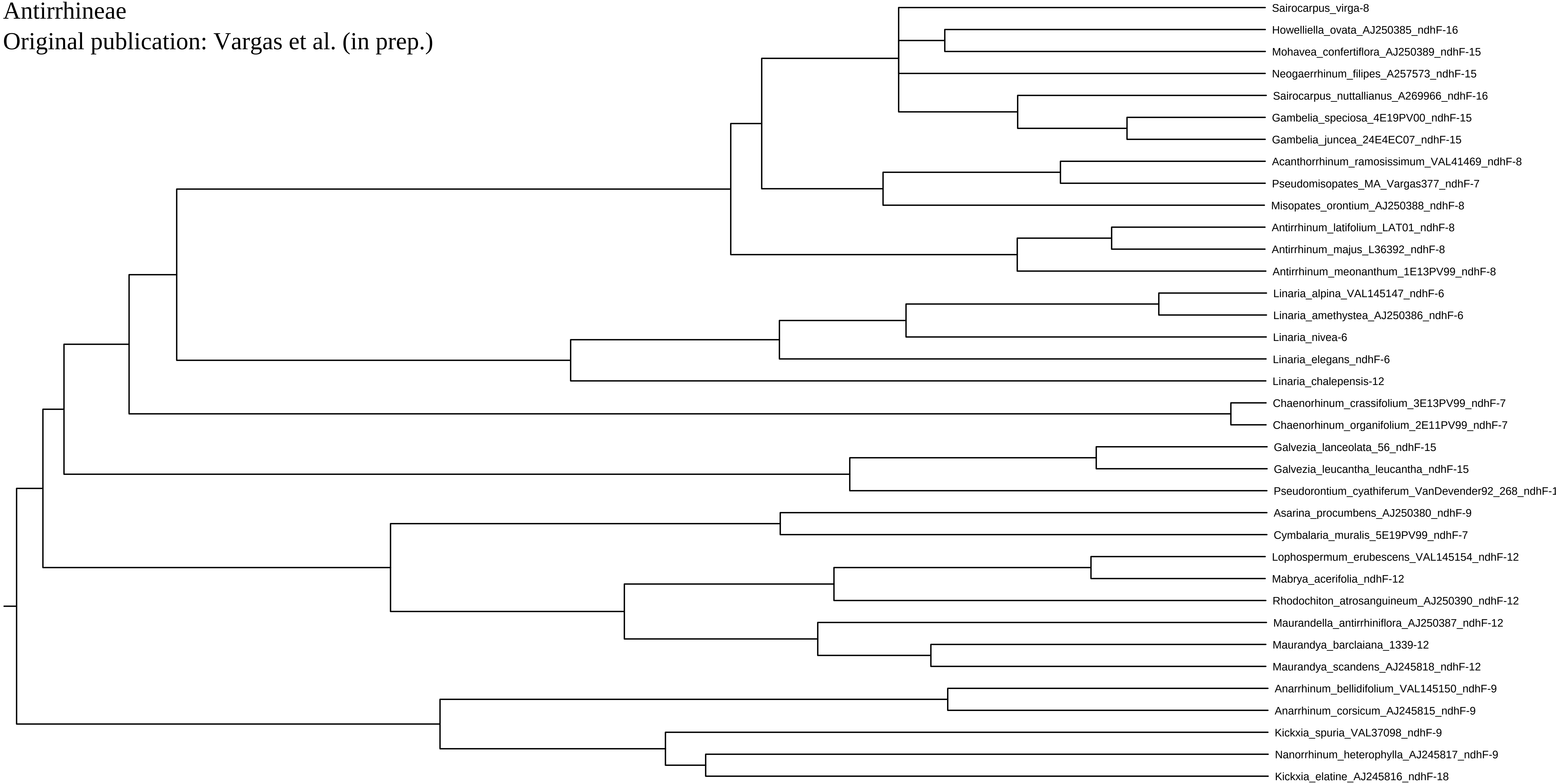

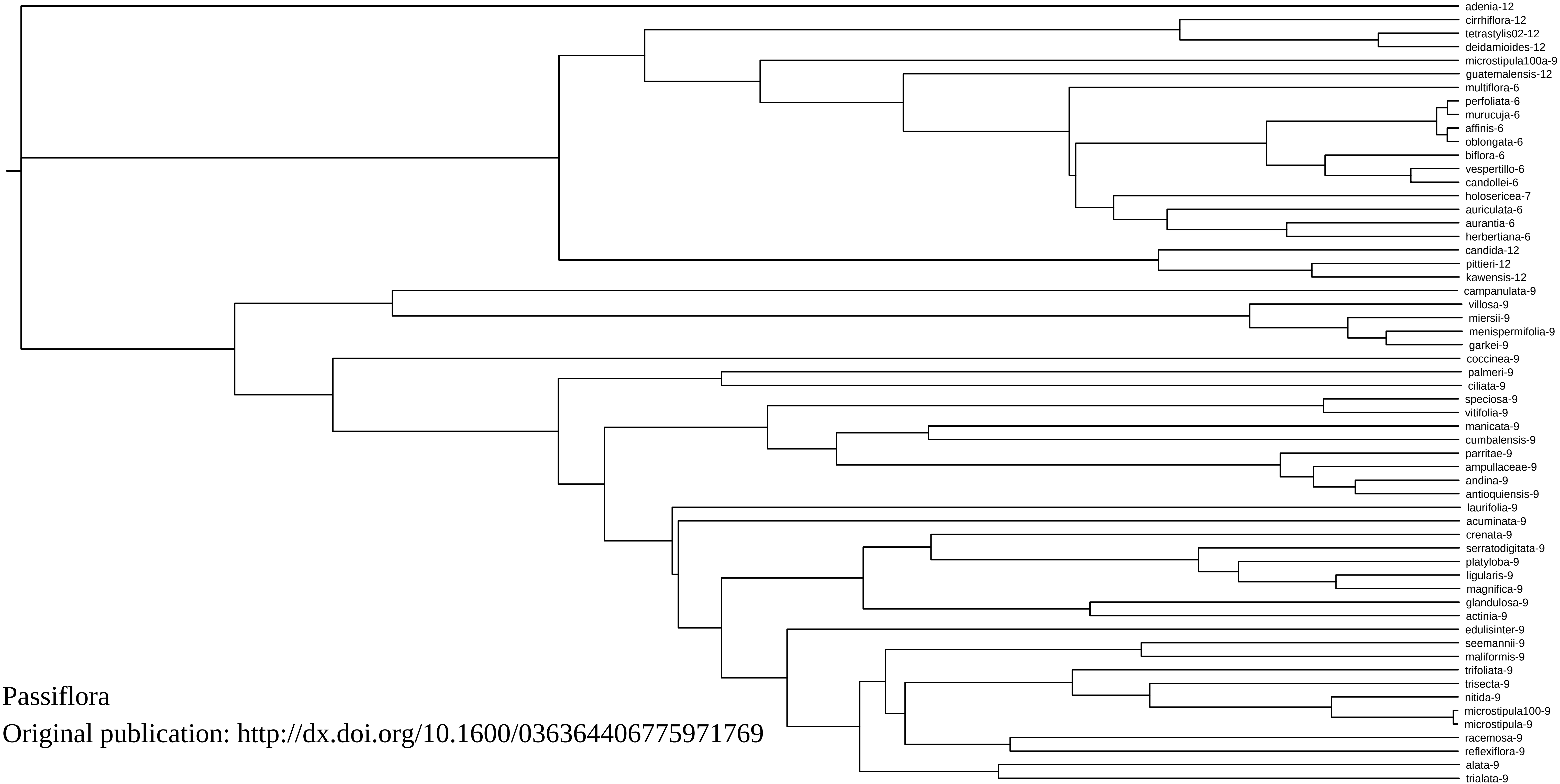

Passiflora

Original publication: http://dx.doi.org/10.1600/036364406775971769

Cistaceae

Original publication: http://dx.doi.org/10.1016/j.ode.2009.01.001

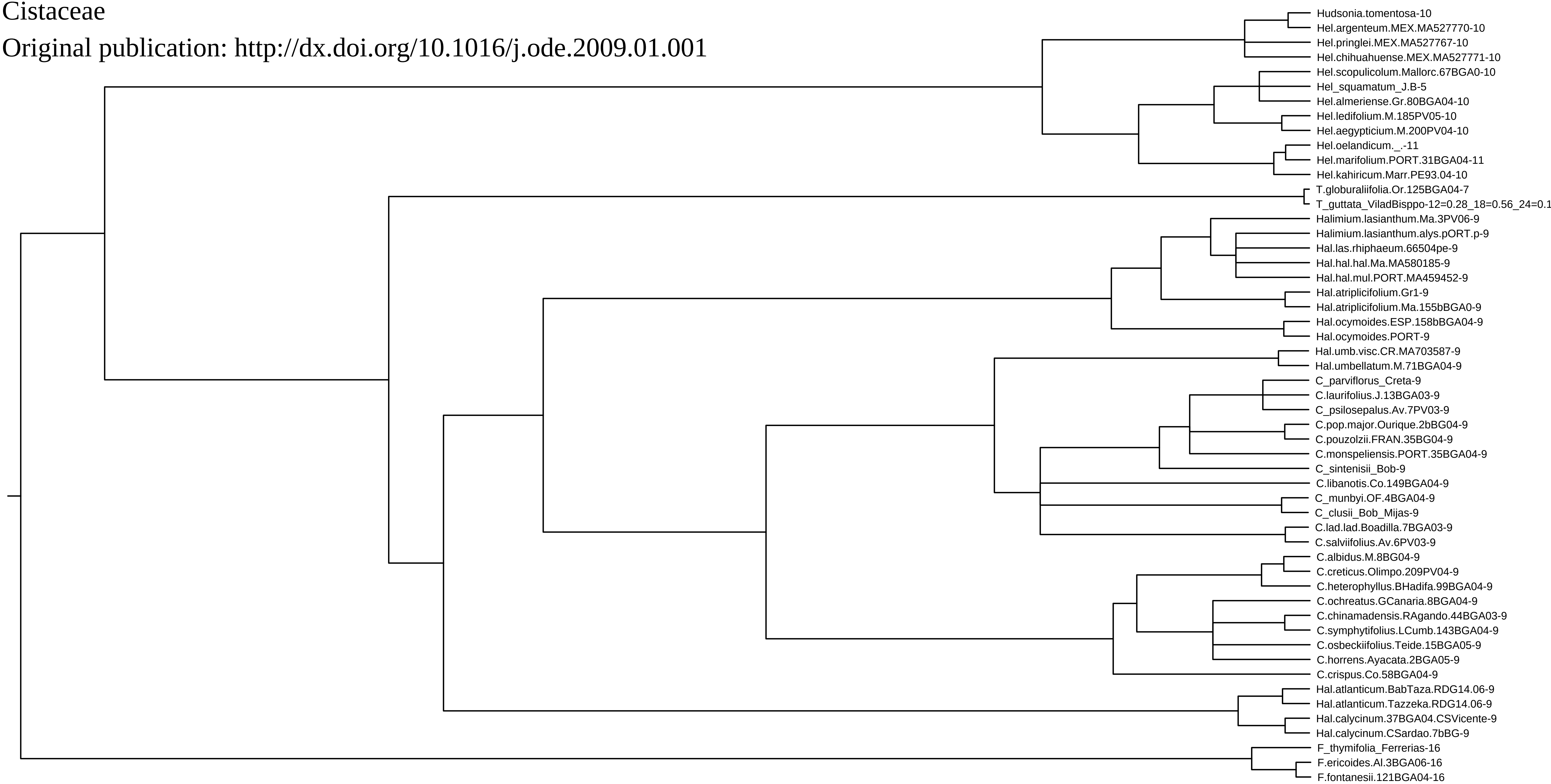

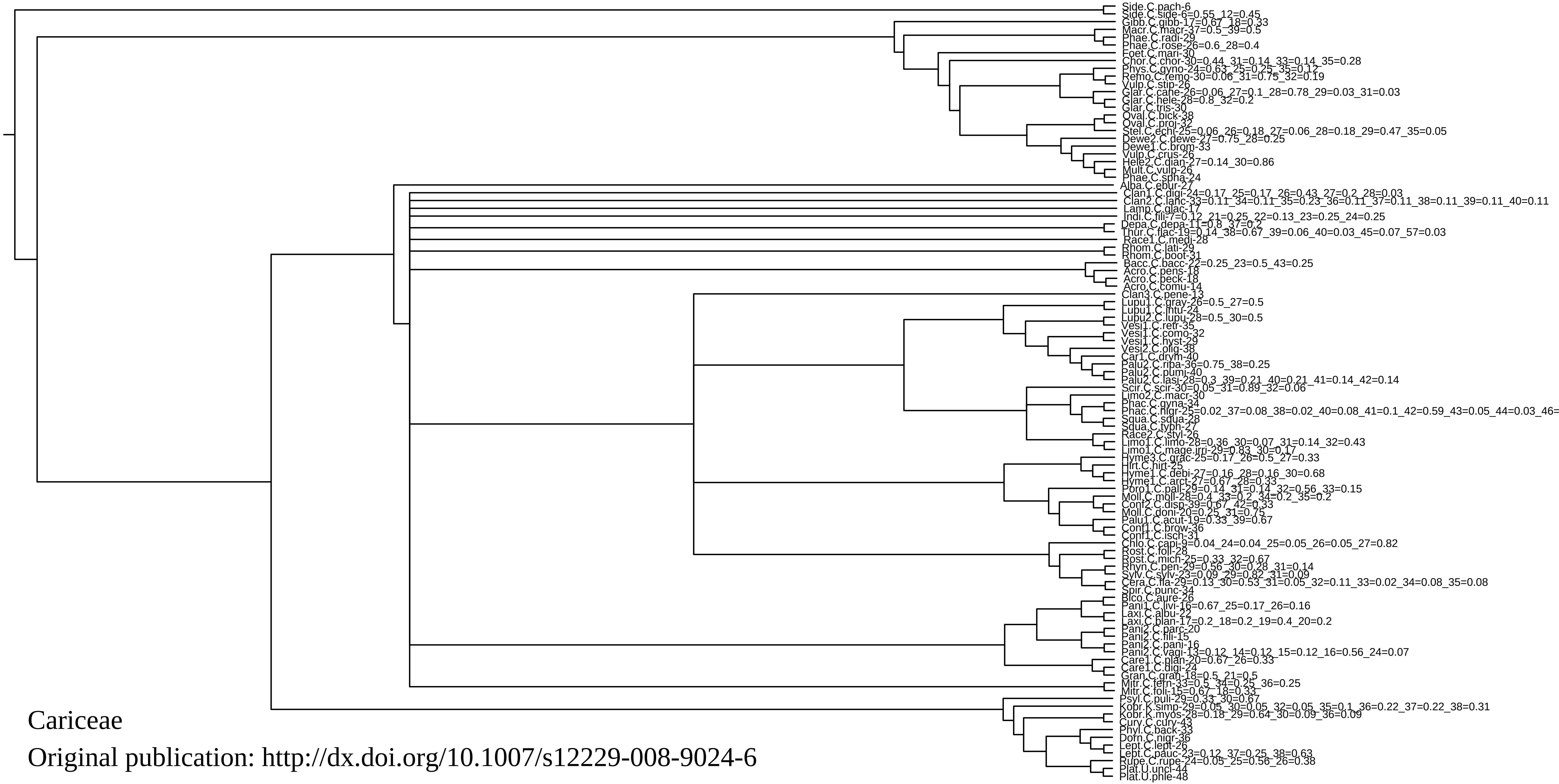

Cariceae

Original publication: http://dx.doi.org/10.1007/s12229-008-9024-6

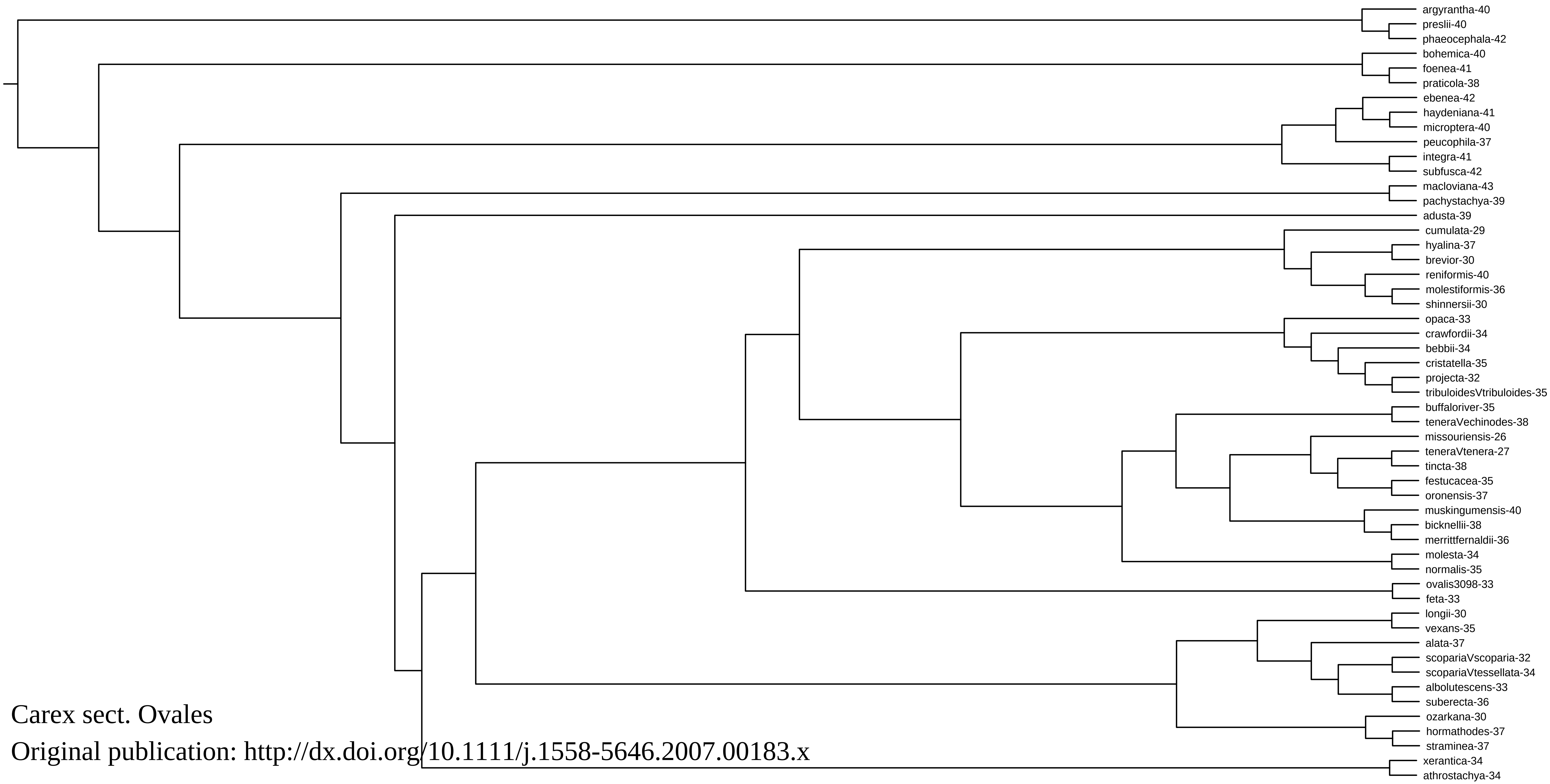

Carex sect. Ovales

Original publication: http://dx.doi.org/10.1111/j.1558-5646.2007.00183.x

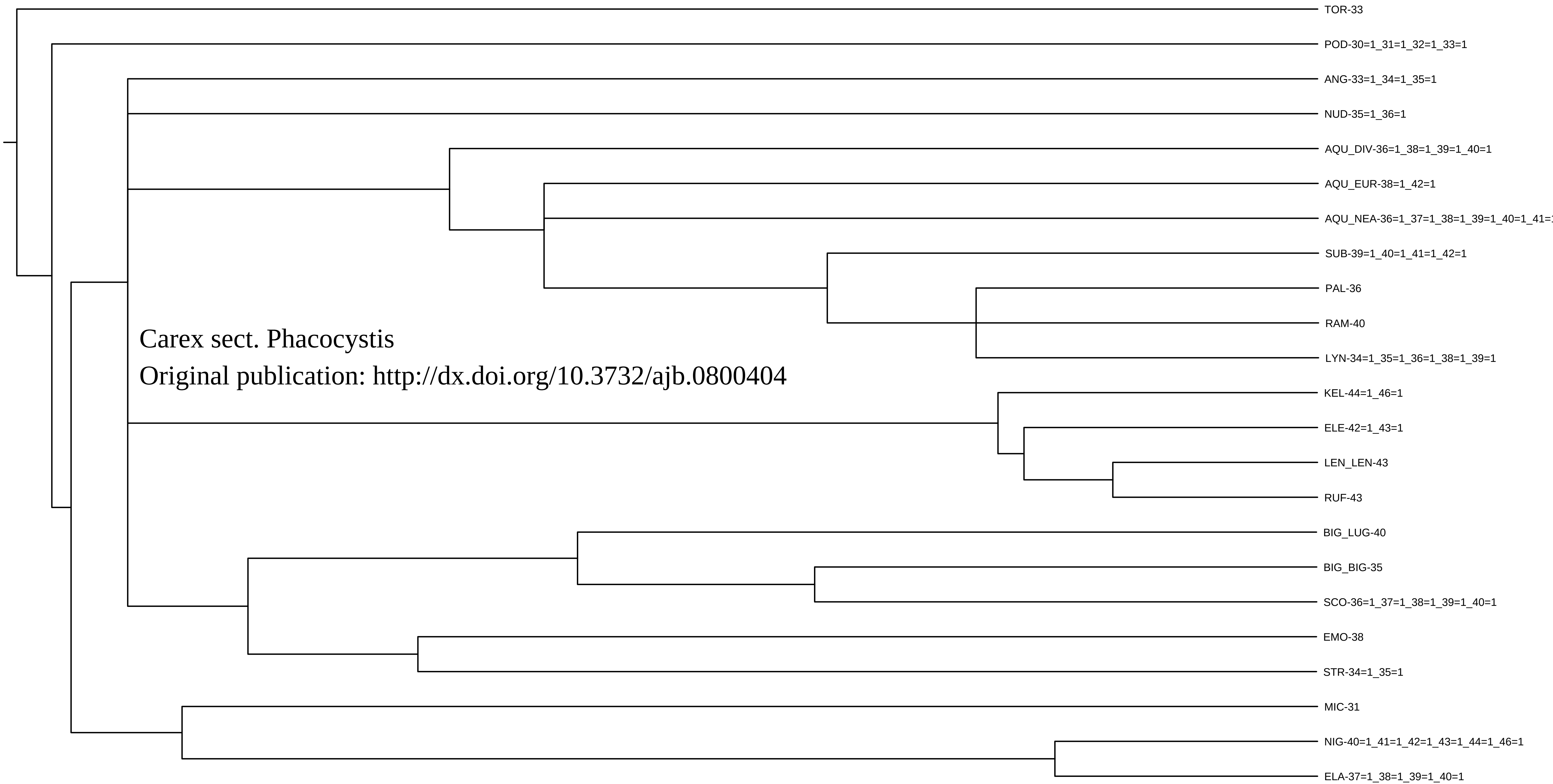
TOR-33
POD-30=1_31=1_32=1_33=1
ANG-33=1_34=1_35=1
NUD-35=1_36=1
AQU_DIV-36=1_38=1_39=1_40=1
AQU_EUR-38=1_42=1
AQU_NEA-36=1_37=1_38=1_39=1_40=1_41=
SUB-39=1_40=1_41=1_42=1
PAL-36
RAM-40
LYN-34=1_35=1_36=1_38=1_39=1
Carex sect. Phacocystis
Original publication: http://dx.doi.org/10.3732/ajb.0800404
KEL-44=1_46=1
ELE-42=1_43=1
LEN_LEN-43
RUF-43
BIG_LUG-40
BIG_BIG-35
SCO-36=1_37=1_38=1_39=1_40=1
EMO-38
STR-34=1_35=1
MIC-31
NIG-40=1_41=1_42=1_43=1_44=1_46=1
ELA-37=1_38=1_39=1_40=1

Carex sect. Spirostachyae

Original publication: http://dx.doi.org/10.3732/ajb.0900134

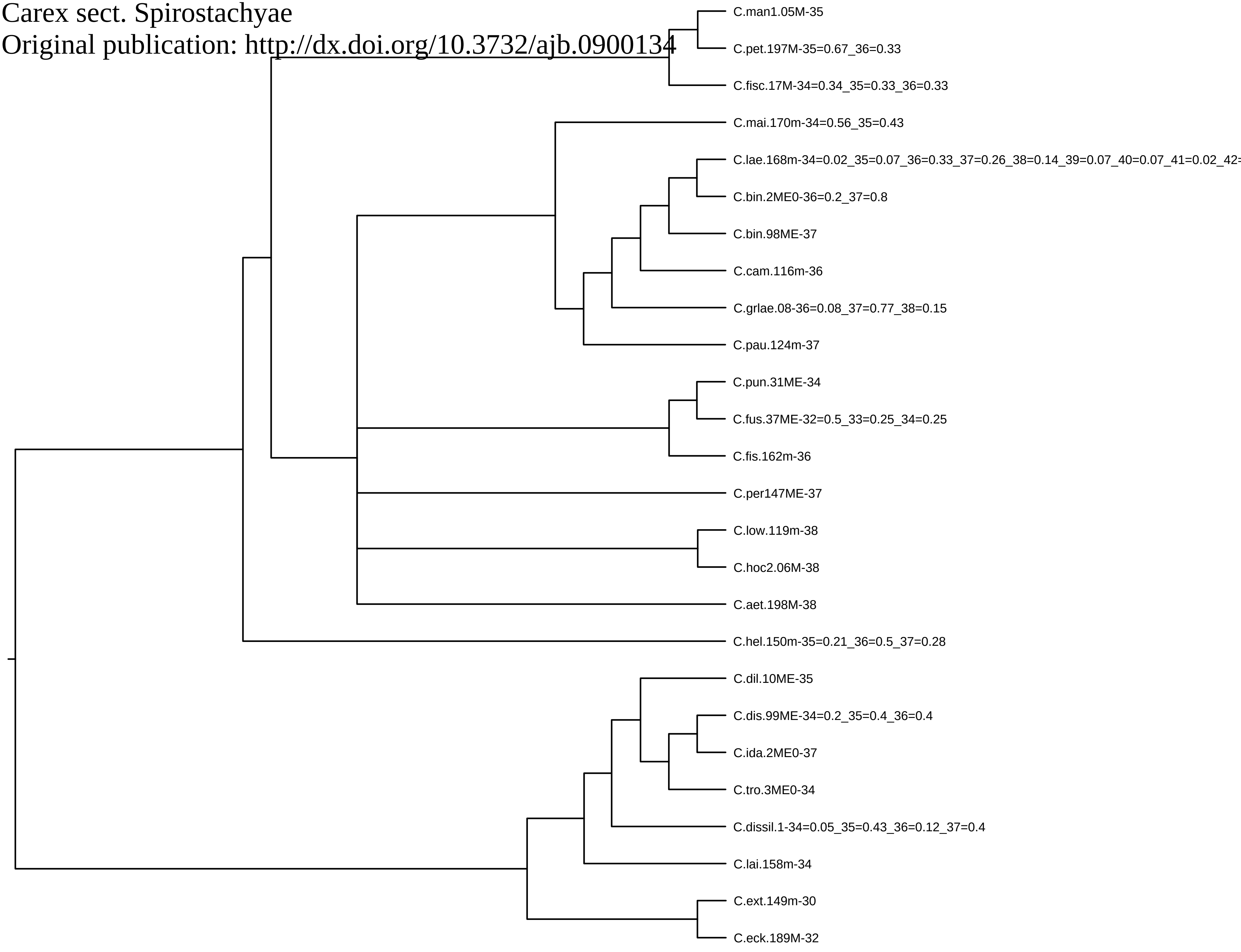

Saxifraga sect. Saxifraga

Original publications: http://dx.doi.org/10.1007/BF00985326

http://dx.doi.org/10.1111/j.1756-1051.2001.tb01340.x

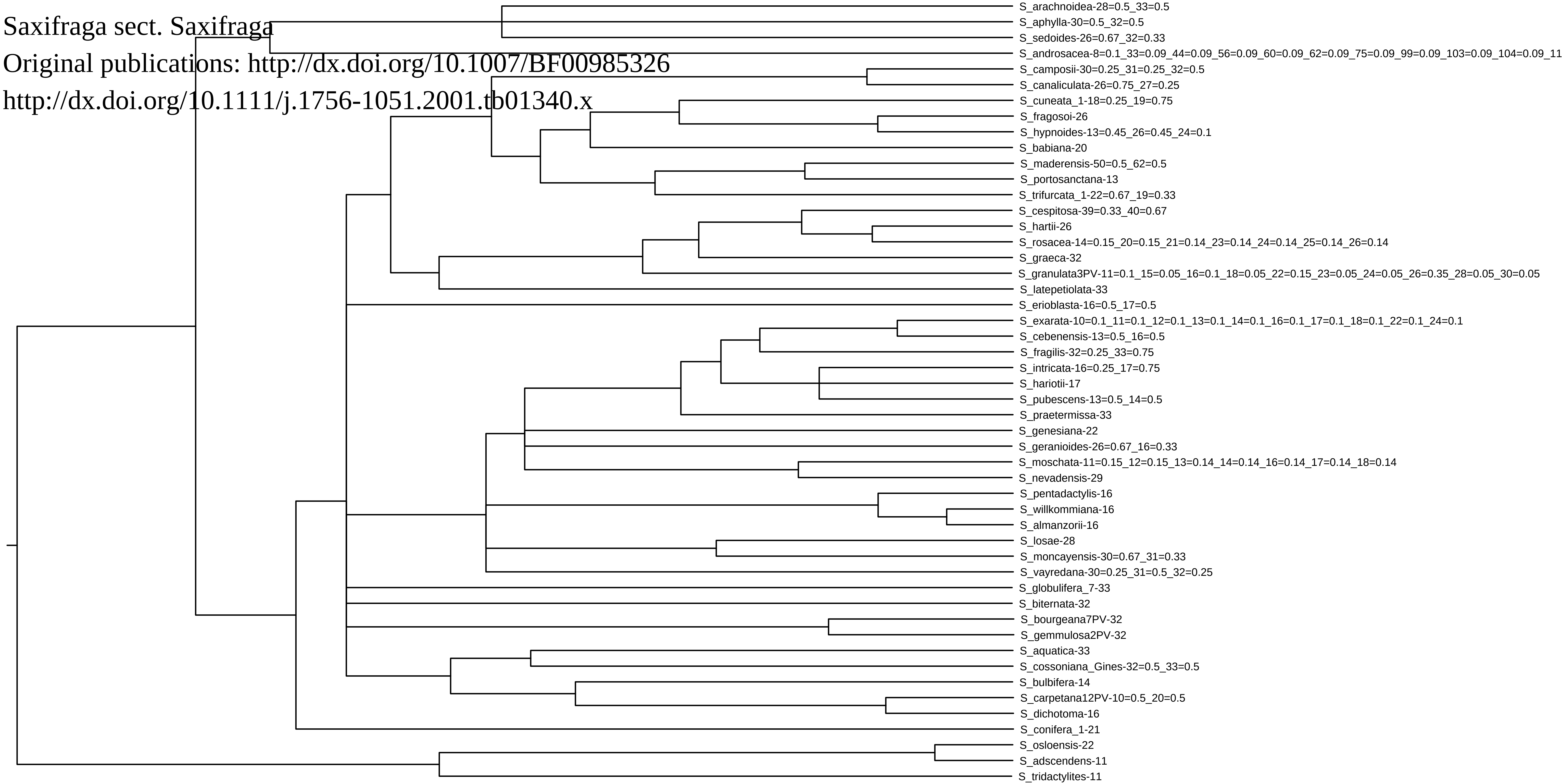

Supplement: Material S1 — Zip file with graphs of phylogenetic trees with haploid chromosome numbers in the tips and phylogentic trees in parenthetical format from chromEvol analyses with inferred mutation events. (ZIP) [file pone.0085266.s003.zip › Supplementary Material 1/Supplementary Material 1 - Graphs.pdf]
